# Syringin exerts anti-breast cancer effects through PI3K-AKT and EGFR-RAS-RAF pathways

**DOI:** 10.1186/s12967-022-03504-6

**Published:** 2022-07-06

**Authors:** Fei Wang, Chong Yuan, Bo Liu, Yan-Fang Yang, He-Zhen Wu

**Affiliations:** 1 Faculty of Pharmacy, Hubei University of Chinese Medicine, Wuhan, 430065 China; 2 Key Laboratory of Traditional Chinese Medicine Resources and Chemistry of Hubei Province, Wuhan, 430061 China

**Keywords:** Syringin, *Acanthopanax senticosus* (Rupr. & Maxim.) Harms, Breast cancer, Hub genes, Bioinformatics, Mechanism of action

## Abstract

**Background:**

Breast cancer (BC) is one of the most common malignant tumors with the highest mortality in the world. Modern pharmacological studies have shown that Syringin has an inhibitory effect on many tumors, but its anti-BC efficacy and mechanism are still unclear.

**Methods:**

First, Syringin was isolated from *Acanthopanax senticosus* (Rupr. & Maxim.) Harms (ASH) by systematic solvent extraction and silica gel chromatography column. The plant name is composed of genus epithet, species additive words and the persons’ name who give its name. Then, the hub targets of Syringin against BC were revealed by bioinformatics. To provide a more experimental basis for later research, the hub genes which could be candidate biomarkers of BC and a ceRNA network related to them were obtained. And the potential mechanism of Syringin against BC was proved in vitro experiments.

**Results:**

Syringin was obtained by liquid chromatography-mass spectrometry (LC–MS), nuclear magnetic resonance (NMR), and high-performance liquid chromatography (HPLC). Bioinformatics results showed that MAP2K1, PIK3CA, HRAS, EGFR, Caspase3, and PTGS2 were the hub targets of Syringin against BC. And PIK3CA and HRAS were related to the survival and prognosis of BC patients, the PIK3CA-hsa-mir-139-5p-LINC01278 and PIK3CA-hsa-mir-375 pathways might be closely related to the mechanism of Syringin against BC. In vitro experiments confirmed that Syringin inhibited the proliferation and migration and promoted apoptosis of BC cells through the above hub targets.

**Conclusions:**

Syringin against BC via PI3K-AKT-PTGS2 and EGFR-RAS-RAF-MEK-ERK pathways, and PIK3CA and HRAS are hub genes for adjuvant treatment of BC.

**Graphical Abstract:**

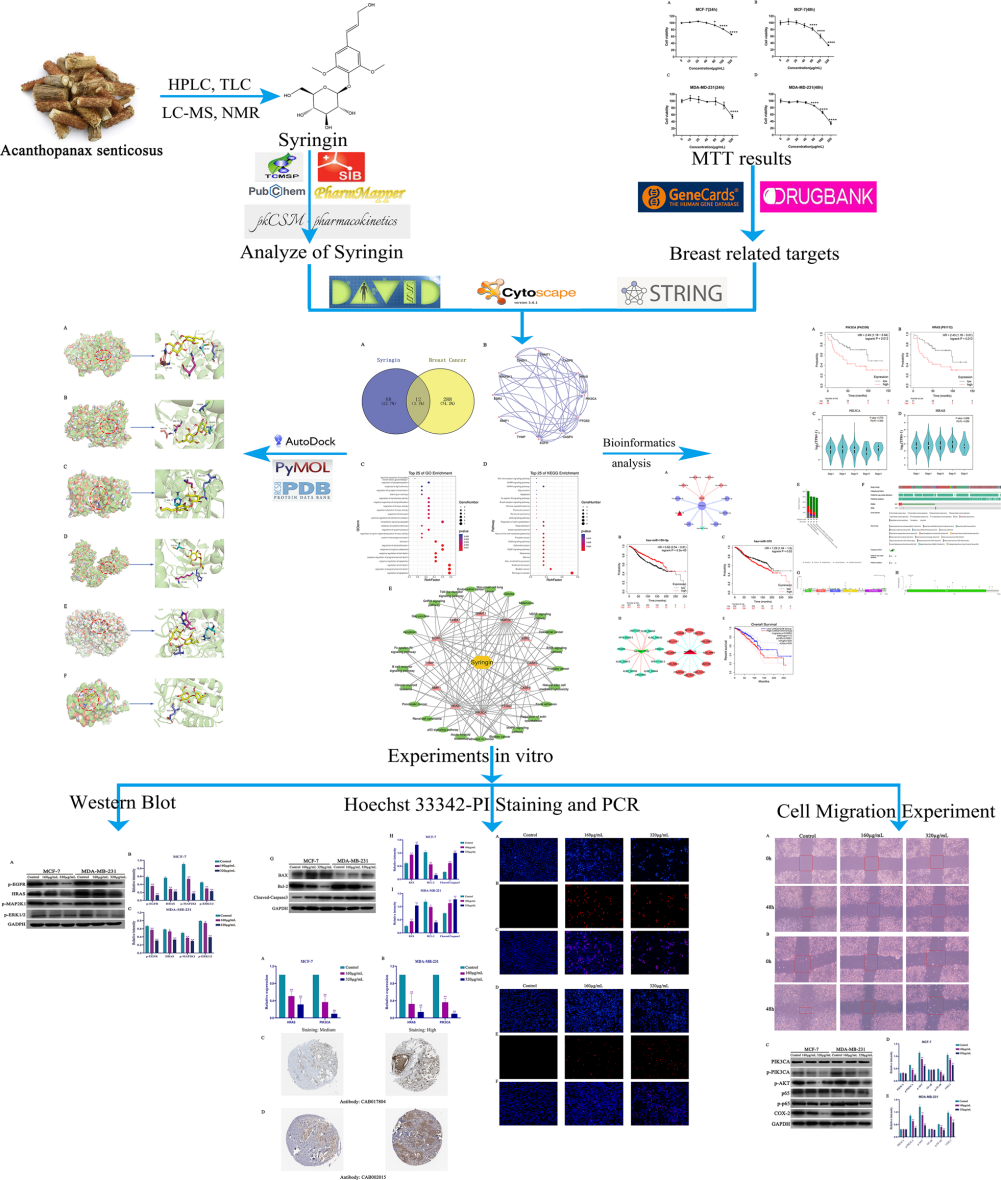

**Supplementary Information:**

The online version contains supplementary material available at 10.1186/s12967-022-03504-6.

## Background

Breast cancer (BC) is one of the most common malignant tumors with the highest mortality in the world and is usually caused by genetic and environmental factors [1]. According to the location, it can be divided into invasive and non-invasive BC. Depending on the type of BC, hormone therapy, targeted therapy, and immunotherapy are the current systemic treatment options alongside conventional chemotherapy. Regardless of the type, stage 0 BC is noninvasive and can be treated with lumpectomy, radiation therapy, or hormone therapy. Treatment of early aggressive stages (I, IIa, IIb) and locally advanced stages (IIIa, IIIb, IIIc) varies depending on whether the tumor expresses estrogen, progesterone, or ERBB2 receptors, but surgical resection remains the most common means. Undeniably, early diagnosis of the disease is critical for effective treatment and positive prognosis, as patients with smaller tumors found at diagnosis have significantly lower mortality rates and higher survival rates [2]. Therefore, it is necessary to develop more effective, cheaper, and safer drugs.

Traditional Chinese medicine (TCM) has a certain history in the treatment of tumors [3]. Due to the strong anti-tumor effect, mild side effects, and other advantages, Chinese herbs have attracted widespread attention [4, 5]. Fu M et al. demonstrated that coptis chinensis and dried ginger herb combination can inhibit the growth of gastric tumors by regulating glucose metabolism by regulating LDHA and SLC2A1 genes [6]. The extract of liquorice can down-regulate CDK4-Cyclin D1 complex and increase CD8^+^ T cell infiltration to exert anti-non-small cell lung cancer effect [7]. According to the theory of TCM [8], the pathogenesis of tumors can be summarized as qi stagnation and blood stasis, heat toxin accumulation, qi and blood deficiency, and meridian stasis. Therefore, TCM advocates “strengthening the body and resisting evil”, that is, supporting the healthy qi of the human body and improving the body’s disease resistance to achieve the purpose of eliminating the tumor. *Acanthopanax senticosus* (Rupr. & Maxim.) Harms (ASH) was recorded in *Shennong’s Classic of Materia Medica* for the first time and was listed as the top grade. The ancient book divides Chinese herbs into top, middle and low grades. The top grade is a life prolonging drug and non-toxic. The middle grade is a disease-preventing and tonifying medicine, which is toxic and non-toxic depending on the dosage. And the lower grade is a medicine for treating and preventing diseases. It is toxic and should not be taken for a long time. According to the records of *Mingyi Bielu*, ASH has the effects of improving the interior, enhancing the body resistance, benefiting essence, and strengthening bones. Modern pharmacological studies have shown that ASH has anti-cancer effects, such as in liver cancer, gastric cancer, and colon cancer [9], and has been applied in many kinds of marketed drugs that approved by the national medical products administration in China. Clinical data show that FuFangBanMaoJiaoNang which contains ASH has a significant effect in the treatment of triple-negative breast cancer, and can effectively inhibit postoperative recurrence and metastasis [10]. The marketed Chinese patent medicine Aidi injection has the functions of softening and decomposing hard lumps, clearing heat and detoxifying, and has been used to treat gastric cancer, breast cancer, and other cancers [11]. As the main component of these clinical drugs, ASH and its extract are reported to induce non-apoptotic cell death through mitochondria related to ROS dependent and independent pathways in BC cells [12]. Syringin, also known as eleutheroside B, is the main active component of ASH. It is a nutritional and pharmaceutical component commonly used for anti-inflammatory and enhancing immunity and has broad application prospects [13]. Previous studies in our laboratory have shown that the methanol extract of ASH significantly decreased the growth of MCF-7 cells, as shown in Additional file 1: Figure S1. And Syringin has inhibitory effects on MDA-MB-231 and MCF-7 [14]. Therefore, it is necessary to study the inhibitory effect of Syringin on BC and its possible mechanism.

Through the comprehensive utilization of system bioinformatics, multi-directional pharmacology, cutting-edge computer science, and information technology, the hub targets in survival, prognosis, stage, and immunity of tumors were screened by 24 databases and 4 software. At the same time, the mechanism of TCM treatment of diseases could be elucidated by the absorption, distribution, metabolism, excretion, and toxicity (ADMET) principle, “components-targets-pathways (C-T-P)” and “Protein–protein interaction (PPI)” networks. These analytical techniques provide great help for further analysis and popularization of TCMs.

In this study, the (3-(45)-dimethylthiahiazo (-z-y1)-35-di-phenytetrazoliumromide) (MTT) assay was used to investigate the effects of Syringin extracted from ASH on the proliferation of MDA-MB- 231 and MCF-7 cells. The two cell lines were identified as the objects of study. Then, the related targets of Syringin against them were screened by system bioinformatics, and the hub genes which could be candidate biomarkers and a ceRNA network of them were obtained. Finally, the results were verified by western blot, real-time PCR, and in vitro experiments.

## Methods

### Isolation and ADMET information collection of Syringin

By using thin-layer chromatography (TLC), HPLC, NMR, and LC–MS, Syringin was isolated and purified from ASH. The extraction and separation process of it is shown in the appendix, and its purity of it was > 98.0%.

The first step of bioinformatics analysis of Syringin is to collect its identity information and analyze the possibility of its development into a drug with ADMET. Traditional Chinese Medicine Systems Pharmacology Database and Analysis Platform (TCMSP) (http://lsp.nwu.edu.cn/tcmsp.php) [15], SwissADME (http://www.swissadme.ch/index.php) [16], pkCSM (http: //biosig.unimelb.edu.au/pkcsm/prediction) databases [17] and PubChem (https://pubchem.ncbi.nlm.nih.gov/) [18] were used to collect the above information.

### Cell culture

MDA-MB-231 and MCF-7 cells were obtained from Hubei University of Chinese Medicine (Wuhan, China). It is worth noting that MCF-7 cells are insulin-dependent in culture. Therefore, we consulted the literature and chose the most commonly used method to culture MCF-7 cells with the high sugar and high serum content of the culture medium they need. The cells were cultured in Dulbecco's modified eagle medium basic (DMEM, 1X, Gibco, MD, USA) supplemented with 12% fetal bovine serum (FBS) (Gibco, MD, USA) and penicillin–streptomycin solution (The concentration of penicillin in cell culture medium was 100 µ/ml, and that of streptomycin was 0.1 mg/ml). And they were incubated at 37 °C under 5% CO_2_ in a carbon dioxide incubator (HHCP-01, Shanghai BAIDIAN Instrument Equipment Co., Ltd, China).

### Effect of syringin on cell proliferation

Firstly, MDA-MB-231 and MCF-7 cells were incubated in different 96-well plates at a density of 6.0 × 10^3^ cells per well. After cell adhesion, each group was given different concentrations of Syringin (0, 20, 40, 80, 160, and 320 µg/mL) for 24 h and 48 h respectively. Then, 20 µL MTT (Sigma, MO, USA) was added to each well, and cells were cultured for another 4 h. After removing the supernatant, 150 µL dimethyl sulfoxide (DMSO) (Sigma) was added to each well and stirred on QB-9001 micropore rapid shaker (Kylin-Bell Lab Instruments Co. Ltd., Jiangsu, China) for 10 min to obtain crystal violet product. Finally, the absorbance of each well was read at 490 nm using a spark 10 m microplate reader (Tecan, Männedorf, Switzerland).

### Candidate genes collection

This study used six databases to screen the targets of Syringin against these two BC cell lines. Firstly, importing the structure of Syringin into SwissTarget Prediction (https://labworm.com/tool/swisstargetprediction) and PharmMapper databases (http://www.lilab-ecust.cn/pharmmapper/submitfile.html) to obtain all targets of Syringin. Then, the species was limited to humans, and the Gene Official Symbol format of targets was obtained via the Retrieve/ID mapping of Uniprot (https://www.uniprot.org/) database. The related genes of these two BC cell lines were obtained with the keyword “Breast cancer” by GeneCards (https://www.genecards.org/) and DrugBank (https://www.drugbank.ca/) databases at the same time. Finally, the Venny 2.1.0 database (https://bioinfogp.cnb.csic.es/tools/venny/) was used to find their intersection, which was the candidate targets.

### Gene ontology and KEGG pathway enrichment

By using Database for Annotation, Visualization, and Integrated Discovery (DAVID) database (https://david.ncifcrf.gov/), the candidate targets related to the Gene Ontology (GO) enrichment and Kyoto Encyclopedia of Genes and Genomes (KEGG) pathway were analyzed. Then, the top 25 KEGG pathways and GO entries (*P* < 0.05) were drawn as bubble plots via the Omicshare database (http://www.omicshare.com) [19].

### Screening of hub genes

Using the plug-in of Cytoscape 3.8.1 software (https://cytoscape.org/download.html), a “C-T-P” network was constructed to analyze the association among them [20]. According to the topological parameters of CytoHubba, the hub genes were selected. Then, using the STRING database (https://string-db.org/) to construct a “PPI” network about the 12 candidate genes [21]. After a comprehensive analysis of the two results, the hub genes were obtained.

### Molecular docking

Semi-flexible molecular docking technology is widely used to study the binding energy between drugs and genes, to verify the interaction sites and amino acid residues between them. AutoDockTools-1.5.6, PyMOL, Chem Draw, RSCBPDB (https://www.rcsb.org/), and PubChem are the most commonly used software or databases for molecular docking [22]. First, importing the hub targets into the RSCBPDB to download their PDB formats, and then download the 3D structure of Syringin through PubChem. After processing with PyMOL and Chem Draw respectively, they were imported into AutoDockTools-1.5.6 to dock. By analyzing the clusters in docking results, the optimal conformations of Syringin and hub genes were obtained according to the binding energy. It is commonly believed that the docking energy is inversely proportional to the interaction force between them, and the threshold is—4 kcal/mol. At the same time, the maximum distance of hydrogen bond between component and protein was set to 2 nm to ensure the accuracy of molecular docking.

Then, the protein–ligand interaction profiler (https://plip.biotec.tu-dresden.de/plip-web/plip/index) and ZBH-Center for Bioinformatics (https://proteins.plus/) databases were used to describe the binding amino acid residues of the components and genes in the original PDB database [23, 24]. Finally, the differences and similarities between them were analyzed.

### Screening of genes significantly related to survival and prognosis of BC patients

Studying the relationship between hub genes and the survival rate of tumor patients is an important basis to judge whether they can be developed as biomarkers. The relationship between hub genes and stage of tumor also provides a valuable reference for the judgment of patients’ condition. Therefore, the Kaplan Meier plotter (http://kmplot.com/analysis/index.php) and gene expression profiling interactive analysis (GEPIA) 2.0 (http://gepia.cancer-pku.cn/index.html) databases were used respectively to explore their relationship [25, 26]. The Kaplan Meier plotter includes data from gene expression omnibus (GEO), METABRIC, and the cancer genome atlas (TCGA) databases. Its purpose was to evaluate the effects of 30 k genes (including mRNA, miRNA, and protein) on the survival rate of 21 cancer types, to find and verify survival biomarkers. And using the “Single gene analysis” module in GEPIA 2.0, the expression of hub genes in different stages in BC was analyzed. Based on 9736 tumors and 8587 normal tissues from TCGA and genotype-tissue expression (GTEX) projects, genes expression in various tumors was analyzed by the ANOVA method (setting |lg FC|< 1 and Q-value < 0.05).

### Investigation the mutation of genes related to survival and prognostic of BC

The mutation of genes plays an important role in the occurrence and development of tumors. It causes changes in gene structure and quantity and results in the loss of gene function. Therefore, it is meaningful to study the mutation of the screened genes related to survival and prognostic of BC. cBioPortal database (http://www.cbiobortal.org) contains data from multiple tumor genome studies. Using the mutation module of it, the mutation of these genes in 9536 samples of 18 studies about BC from TCGA, MSKCC, MSK and METABRIC databases and 8 institutions including Broad and British Columbia were analyzed [27].

### Construction of “mRNA-miRNA-lncRNA” network

LncRNA indirectly inhibits the negative regulation of miRNA on genes by competing with miRNA to bind the 3'-UTR of mRNA. Therefore, exploring the relationship between these three is conducive to a more comprehensive exposition of the mechanism of Syringin against these two BC cell lines. Using miRTarBase (http://mirtarbase.mbc.nctu.edu.tw) and miRWalk (http://mirwalk.umm.uni-heidelberg.de/) databases to screen miRNAs corresponding to biomarkers [28, 29]. After the miRNAs significantly associated with prognosis of patients with BC were obtained via Kaplan Meier plotter and GEPIA 2.0, TarBase v.7 (https://doi.org/10.1093/nar/gkx1141) and lnCAR (https://lncar.renlab.org/explorer) databases were used to screen lncRNA significantly associated with miRNA (score threshold was 0.9) [30, 31]. Then, the relationship between lncRNA and prognosis of BC patients was analyzed by “Survival Analysis” in GEPIA 2.0 databases. Finally, a “mRNA-miRNA-lncRNA” (ceRNA) network is constructed by Cytoscape 3.8.1 software.

### Plate cloning experiment

The 6-well plates were divided into three groups, and 2.0 × 10^3^ cells (MDA-MB-231 and MCF-7) were added to each well. After adherence, added 2 mL of Syringin with the concentration of 0, 160, 320 µg/mL to each well respectively. 48 h later, replaced the medium in each well. On the 15th day, the medium of each well was discarded and the cells were fixed with 4% polyoxymethylene for 15 min. After washing with PBS 3 times, 1.5 mL crystal violet was added to each well. 20 min later, the 6-well plates were cleaned with flowing water until the background was clear and photos were taken.

### Hoechst 33,342/PI staining experiment

The 6-well plates were divided into three groups, and 5.0 × 10^5^ cells (MDA-MB-231 and MCF-7) were added to each well. After adherence, added 2 mL of Syringin with the concentration of 0, 160, 320 µg/mL was added for 48 h, respectively. Then, the cells were fixed with 4% polyoxymethylene for 15 min. After washing with PBS 3 times, 1.0 mL Hoechst 33342 (Beyotime, 8 µg/mL) to each well for 15 min at 37 °C. Then, added 1.0 mL propidium iodide (PI) (Biosharp, 20 µg/mL) to each well at 4 °C for 10 min, and observed the cells with a fluorescence microscope.

### Cell migration experiment

The 6-well plates were divided into three groups, and 5.0 × 10^5^ cells (MDA-MB-231 and MCF-7) were added to each well. After adherence, scratched the bottom of each well with the tip of a 1 mL sterilized pipette. Added 2 mL of Syringin with the concentration of 0, 160, 320 µg/mL to each well for 48 h. Then the migration of cells in each well was observed by microscope at different times.

### Analysis of Western blot

MDA-MB-231 and MCF-7 cells were inoculated in 6-well plates at the density of 5.0 × 10^5^ cells/well and treated with Syringin (0, 160, 320 µg/mL) for 48 h. Then, the radio-immunoprecipitation assay (RIPA) lysis kit (BL504A, Biosharp, Anhui, CHINA) was mixed with Phenylmethanesulfonyl fluoride (PMSF) (BL504A, Biosharp, Anhui, CHINA) and PI (BL615A, Biosharp, Anhui, CHINA) for cell lysis. After 12,000 rpm centrifugation for 10 min, the total proteins of the whole cell lysate were quantified by a BCA protein assay kit (69,107,317, Biosharp, Anhui, CHINA). The same total proteins were separated on 10% SDS-PAGE gel electrophoresis (AS1012, ASPEN, CHINA) and then electrophoretically transferred to a nitrocellulose membrane by electrophoresis (DYY-6C, Beijing, CHINA). After sealing with 5% BSA at room temperature for 1.0 h, the membranes were washed 3 times and incubated with 13 primary antibodies respectively at 4 °C overnight. The primary antibodies include GAPDH (PMK042M, biopm, Wuhan, China), p-EGFR (CY5596, Abways, Shanghai, China), HRAS (CY5223, Abways, Shanghai, China), p-p65 (CY6367, Abways, Shanghai, China), PIK3CA (CY5355, Abways, Shanghai, China), Bax (AY0553, Abways, Shanghai, China), p-PIK3CA (AB182651, Abcam, Shanghai, China), PTGS2 (12282S, CST, Shanghai, China), p-MAP2K1 (67,873–1-Ig, PTG, Beijing, China), p-ERK1/2 (28,733–1-AP, PTG, Beijing, China), BCL-2 (A0208, Abclonal, Wuhan, China), p65 (GB11997, Google, Wuhan, China), Cleaved-Caspase3 (AF7022, Affinity, Jiangsu, China).The next day, washed the membranes with tris buffered saline + Tween (TBST) 5 times for 30 min. Fresh ECL (170–5060, Bio-Rad, USA) mixed solution was added to the protein side of the membranes and detected by luminescence. The film was scanned and archived, and the optical density of the band was analyzed by the AlphaEaseFC (Alpha Innotech, USA). Using the Image J software to convert the protein image to grayscale image and remove the influence of its background. And then taking the internal parameters as the base, frame each strip, calculate its relative gray value, and visualize the above results in GraphPad Prism 7.0.

### Real-time PCR and the human protein atlas analysis

Total RNA was extracted from each group of cells by Trizol Reagent (15,596,018, Invitrogen, California, USA) according to the manufacturer’s guidelines. The gene primers were designed and synthesized by PINUOFEI Biotechnology Co., Ltd (Wuhan, CHINA). The relevant primer information is shown in Table 1. Then, using FastStart Universal SYBR Green Master (04,913,914,001, Roche, Basel, Switzerland) and SLAN^R^ Real-Time PCR (Shanghai Hongshi Medical Instrument Co., Ltd, CHINA) to determine the expression of mRNAs. The following thermal cycling conditions were adopted: pre-denaturation at 95 °C for 10 min, denaturation at 95 °C for 15 s, denaturation at 60 °C for 60 s (40 cycles). The following condition of the melting curve was used: 60 °C → 95 °C, 1 °C was increased every 20s. GAPDH was used as an internal reference and the 2-ΔΔCT method was used for relative quantification. At the same time, using the Human Protein Atlas database (https://www.proteinatlas.org/) to analyze the gene expression in clinical tumor pathology and normal tissues [32].

**Table 1:** Primers for the hub targets

Primer name	Sequence (5′ to 3′)
Homo GAPDH-F	TCAAGAAGGTGGTGAAGCAGG
Homo GAPDH-R	TCAAAGGTGGAGGAGTGGGT
Homo PIK3CA-F	CGAGTGGTTGGGCAATGAAA
Homo PIK3CA-R	AATGCTTTACTTCGCCGTCC
Homo HRAS-F	TGAGGAGCGATGACGGAATA
Homo HRAS-R	GTATCCAGGATGTCCAACAG

### Statistical analysis

In this study, the significant data of KEGG and GO enrichment were screened according to *P* < 0.05 of students’ t-test. Kaplan–Meier survival plot was used to calculate the hazard ratio of 95% confidence intervals and log-rank P-value. ANOVA was used to analyze the relationship between the hub gene and BC stage in GEPIA 2.0 database, and the thresholds of | log2fc | and Q values were 1 and 0.05 respectively. CBioPortal database was used to obtain information about mutations in hub genes with Z-score. GraphPad Prism 7.0 software (Graph PadSoftware Inc., San Diego, CA) was used for in vitro experiments that were repeated three times, expressed as the mean ± SD, and *P* < 0.05 was considered significant.

## Result

### Information about Syringin

**Fig. 1:**
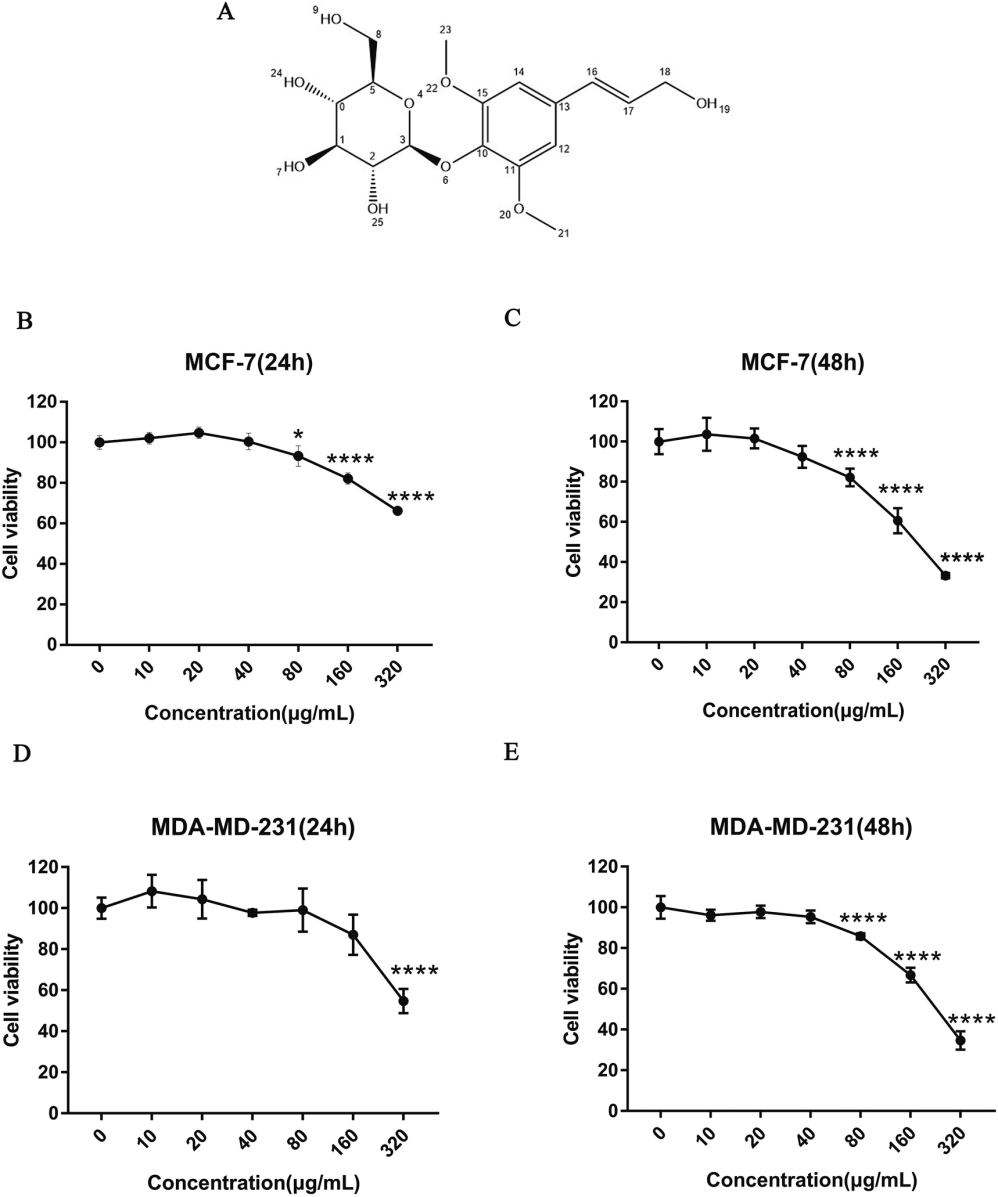
Effect of Syringin on the cancer cells at different times. *P < 0.05 and ****P < 0.001. **A** Structure of Syringin. **B**, **C** MCF-7. **D**, **E** MDA-MB-231

As shown in Additional file 1: Figures S3, S4, the structure of Syringin was identified by using TLC, HPLC, LC–MS, and NMR. Its structural formula is shown in Fig. 1A and the purity of it was over 98% (Additional file 1: Figure S2). The specific separation process and identification results were shown in the supplementary materials. To determine the identity of Syringin, TCMSP and PubChem were used to collect the Molecule CID and Canonical SMILES of it. ADMET results showed that Syringin had low water solubility, intestinal epithelial permeability (Caco-2) permeability, and skin permeability, but its oral bioavailability was very good. Interestingly, it was used as a substrate for PGP rather than an inhibitor of P-glycoprotein, which was an advantage of its development as a drug. The results of VDS and fraction unbound showed that Syringin could be evenly distributed in the body and effectively combined with serum protein to spread to other tissues or organs. Although blood–brain barrier (BBB) and central nervous system (CNS) permeability results failed to show their ability to cross the blood–brain barrier, this didn’t affect our study of its effect against BC. Cytochrome P450 is an important detoxification enzyme in the liver. To investigate whether Syringin was the inhibitor of P450, a drug metabolism study was conducted by pkCSM database. As shown in Table 2, the results confirmed that Syringin was not a matrix or inhibitor of P450, which provided a reference for our later research. The results of total clearance and renal OCT2 substrate showed that Syringin could maintain the drug concentration at a stable rate and had good bioavailability. The results of toxicity studies showed no toxicity and side effects on the liver and skin, and the max accelerated dose (human) of it was higher than the threshold of 0.477 (log mg/kg/day). The results of oral rat acute toxicity (LD 50) and oral rat chronic toxicity (LOAEL) also confirmed that the dose of long-term toxicity was very high, so it was not toxic. Taken together, Syringin conformed to the “Rule of Five”, and it had the potential to be developed as a drug. All the information is shown in Table 2.

**Table 2:** Information about Syringin

Properties	Parameters	Syringin
Identity information	Molecule ID	MOL000347
	PubChem CID	5316860
	Canonical SMILES	COC1=CC(=CC(=C1OC2C(C(C(C(O2)CO)O)O)O)OC)C=CCO
Absorption	Water solubility	-2.148
	Caco2 permeability	0.229
	Intestinal absorption (human)	44.025
	Skin Permeability	-2.758
Distribution	P-glycoprotein substrate	Yes
	P-glycoprotein I inhibitor	No
	P-glycoprotein II inhibitor	No
	VDss (human)	-0.209
	Fraction unbound (human)	0.467
	BBB permeability	-1.25
	CNS permeability	-3.909
Metabolism	CYP2D6 substrate	No
	CYP3A4 substrate	No
	CYP1A2 inhibitor	No
	CYP2C19 inhibitor	No
	CYP2C9 inhibitor	No
	CYP2D6 inhibitor	No
	CYP3A4 inhibitor	No
Excretion	Total Clearance	0.215
	Renal OCT2 substrate	No
Toxicity	AMES toxicity	No
	Max. tolerated dose (human)	0.89
	hERG I inhibitor	No
	hERG II inhibitor	No
	Oral Rat Acute Toxicity (LD50)	1.83
	Oral Rat Chronic Toxicity (LOAEL)	3.718
	Hepatotoxicity	No
	Skin Sensitisation	No
	*T. Pyriformis* toxicity	0.285
	Minnow toxicity	5.982
Drug likeness	Lipinski	Yes; 0 violation
	Veber	Yes
	Muegge	Yes
	Bioavailability Score	0.55
	Rule of Five	Suitable

### Syringin decreased the growth of MDA-MB-231 and MCF-7 cells

MTT results showed that Syringin significantly decreased the growth of the two kinds of cells in a time and dose-dependent manner (*P* < 0.05 and *P* < 0.01). And the half maximal inhibitory concentration (IC 50) of Syringin is 207.9 µg/mL for MCF-7 and 228.8 µg/mL for MDA-MB-231 at 48 h (Fig. 1B–G). Therefore, these two cell lines became the main subjects of this study.

### Candidate genes collection

**Fig. 2:**
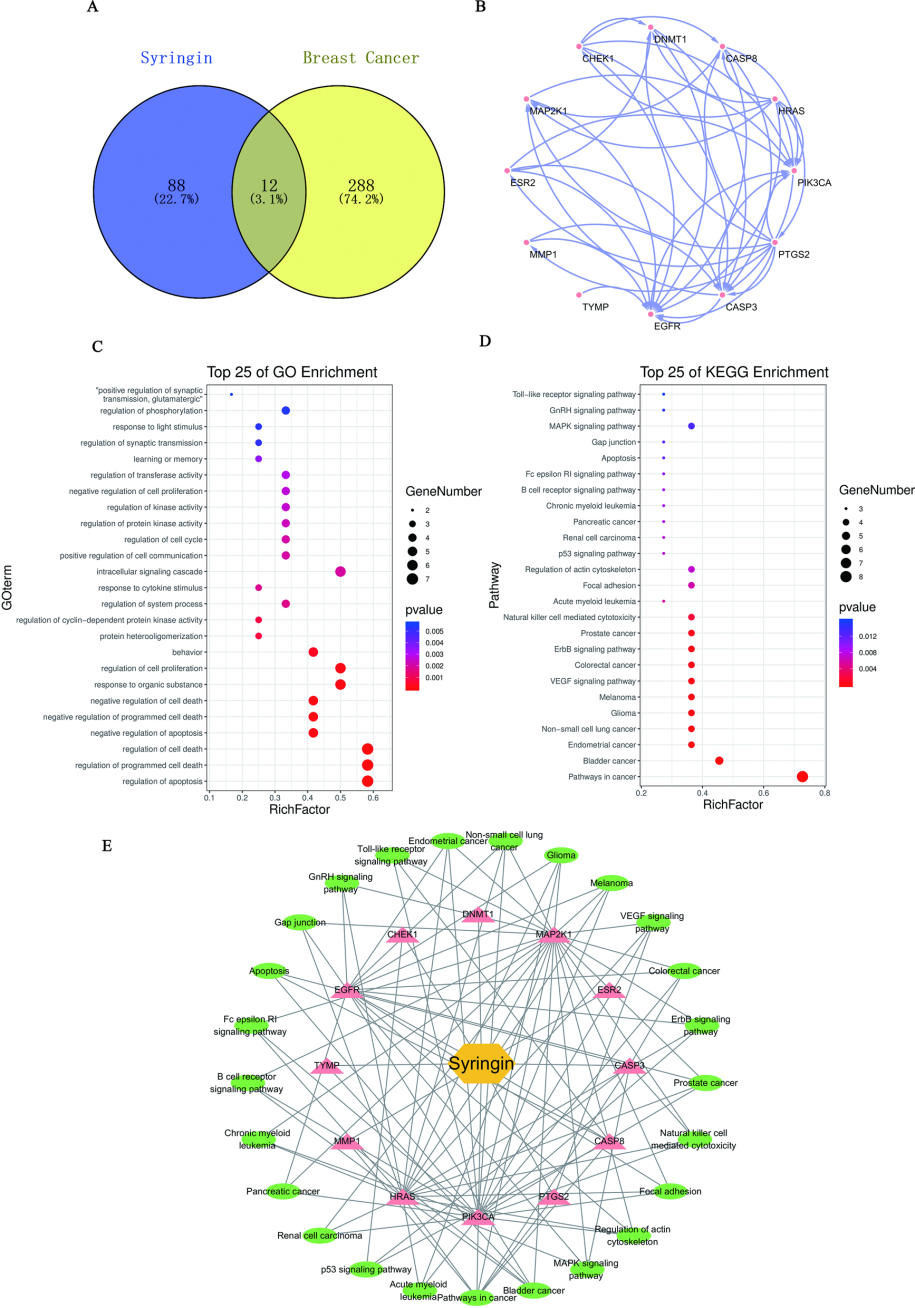
The results of network pharmacology. **A** The overlapping targets of Syringin and BC. **B** A PPI network of overlapping targets. **C** The top 25 of GO enrichment entries. **D** The top 25 of KEGG pathways. **E** A “C-T-P” network

Genes screened from Gene Cards and Drug Bank were analyzed by table tool, and 300 top-ranked genes were obtained. After intersecting them with the genes of Syringin by Venn 2.1.0 database, 12 overlapping genes were analyzed and identified as candidate genes, as shown in Table 3 and Fig. 2A.

**Table 3:** Candidate targets of Syringin anti-BC

Gene Official symbol	Gene names
HRAS	HRas proto-oncogene
MMP1	Matrix metallopeptidase 1
TYMP	Thymidine phosphorylase
EGFR	Epidermal growth factor receptor
CHEK1	Checkpoint kinase 1
DNMT1	DNA methyltransferase 1
MAP2K1	Mitogen-activated protein kinase kinase 1
ESR2	Estrogen receptor 2
CASP3	Caspase 3
CASP8	Caspase 8
PTGS2	Prostaglandin-endoperoxide synthase 2
PIK3CA	Phosphatidylinositol-4,5-bisphosphate 3-kinase catalytic subunit alpha

### Gene ontology and KEGG pathway enrichment analysis

GO and KEGG pathways enrichment analysis of the 12 candidate genes were obtained via the KOBAS 3.0 and DAVID 6.8.1 databases. The results included 29 GO entries (*P* < 0.05), and the top 10 GO enrichment processes were related to positive regulation of ERK1 and ERK2 cascade, regulation of cell proliferation and execution phase of apoptosis, positive regulation of production of miRNAs involved in gene silencing by miRNA, and so on, as shown in Fig. 2C. These suggested that Syringin participated in the above process against these two BC cell lines. In particular, many processes were associated with the proliferation and apoptosis of tumors. In addition, 57 pathways directly or indirectly associated with the occurrence and development of cancer were obtained (*P* < 0.05). The first 25 pathways were obtained by P-value sorting, as shown in Fig. 2D. And 17 of them, including Pathways in cancer, MicroRNAs in cancer, ErbB signaling pathway, Prolactin signaling pathway were directly related to cancer. However, which pathway Syringin acts on needs further analysis and verification.

### “PPI” and “C-T-P” network construction and analysis

Downloaded the TSV format of the “PPI” network from STRING and imported Cytoscape 3.8.1 for visual analysis (Fig. 2B). Using plug-ins, their degrees were analyzed. The degree of EGFR (degree = 11), Caspase 3 (degree = 10), PTGS 2 (degree = 7), HRAS (degree = 8), PIK3CA (degree = 8) and MAP2K1 (degree = 7) were all greater than the median degree (degree = 6). It indicated that they played a more important role in these 12 genes.

As Fig. 2E, constructing a “C-T-P” network that contains 38 nodes (Syringin, 12 targets, and 25 pathways) and 104 lines to analyze their relationship. And using the different colors and shapes to distinguish them. The yellow hexagon represented Syringin, the pink triangle represented candidate targets, and the green circle represented KEGG pathways. And using the lines to show the correlation between them. Obviously, Syringin interacted with different targets and pathways, which was consistent with the concept of multi-targets and multi-pathways synergistic treatment of diseases in TCM. By using CytoHubba to analyze the results, it was not hard to find out that MAP2K1 (degree = 24), PIK3CA (degree = 21), HRAS (degree = 20), EGFR (degree = 15), and Caspase 3 (degree = 7) were all greater than the median degree (degree = 5). Combining the results of the “PPI” network, MAP2K1(degree = 30), PIK3CA (degree = 29), HRAS (degree = 28), EGFR (degree = 26), Caspase 3 (degree = 17) and PTGS 2 (degree = 12) were the hub genes of Syringin in the treatment of MDA-MB-231 and MCF-7 cells.

### Analysis of molecular docking

Using molecular docking, the 6 genes were found to have the strong binding ability with Syringin. To further understand the docking amino acid residues between genes and Syringin, use the automatic grid algorithm to construct a cube containing the largest volume of ligand binding sites and set up 20 potential docking forms. Then, by the step of “transformations > play, ranked by energy”, the conformation results were analyzed. Generally, the RMSD values less than 2.0 Å were classified into the same cluster. According to the principle of docking, the conformation with the lowest binding energy between each other was selected as the best one. Finally, the corresponding genes’ residues were visualized as rods by PyMOL, and different residues were represented with different colors. The results of specific docking are shown in Table 4.

**Table 4:** Docking scores and bonds of the docked inhibitors against proteins

Component	PDB ID	Scores (kcal/mol)	Bonds formed between functional groups of component and protein residues		
			Functional groups	Protein residues	Bond
	5F19	− 7.68	2 (O) 11 (O) 19 (O)	A: HIS 39 A: CYS 47 A: ARG 469	H-bond H-bond H-bond
	7JHP	− 6.53	9 (O) 7 (O) 9 (H)	A: ASP 33 A: LYS 147 A: ASP 119	H-bond H-bond H-bond
*Syringin*	6DUK	− 6.25	19 (O) 20 (O) 24 (O) 19 (H) 24 (H)	A: CYS 797 B: LYS 737 B: LYS 739 A: ASP 800 B: LYS 739	H-bond H-bond H-bond H-bond H-bond
	3ZIM	− 5.62	9 (O) 7 (O) 19 (O) 7 (H) 19 (H)	A: HIS 670 A: CYC 838 A: ALA 758 A: CYS 838 A: ASN 756	H-bond H-bond H-bond H-bond H-bond
	3VVH	− 5.13	9 (O) 20 (O) 7 (H)	B: LYS 205 B: HIS 145 B: CYS 121	H-bond H-bond H-bond
	2H5I	− 4.66	7 (O)	B: GLU 248	H-bond

**Fig. 3:**
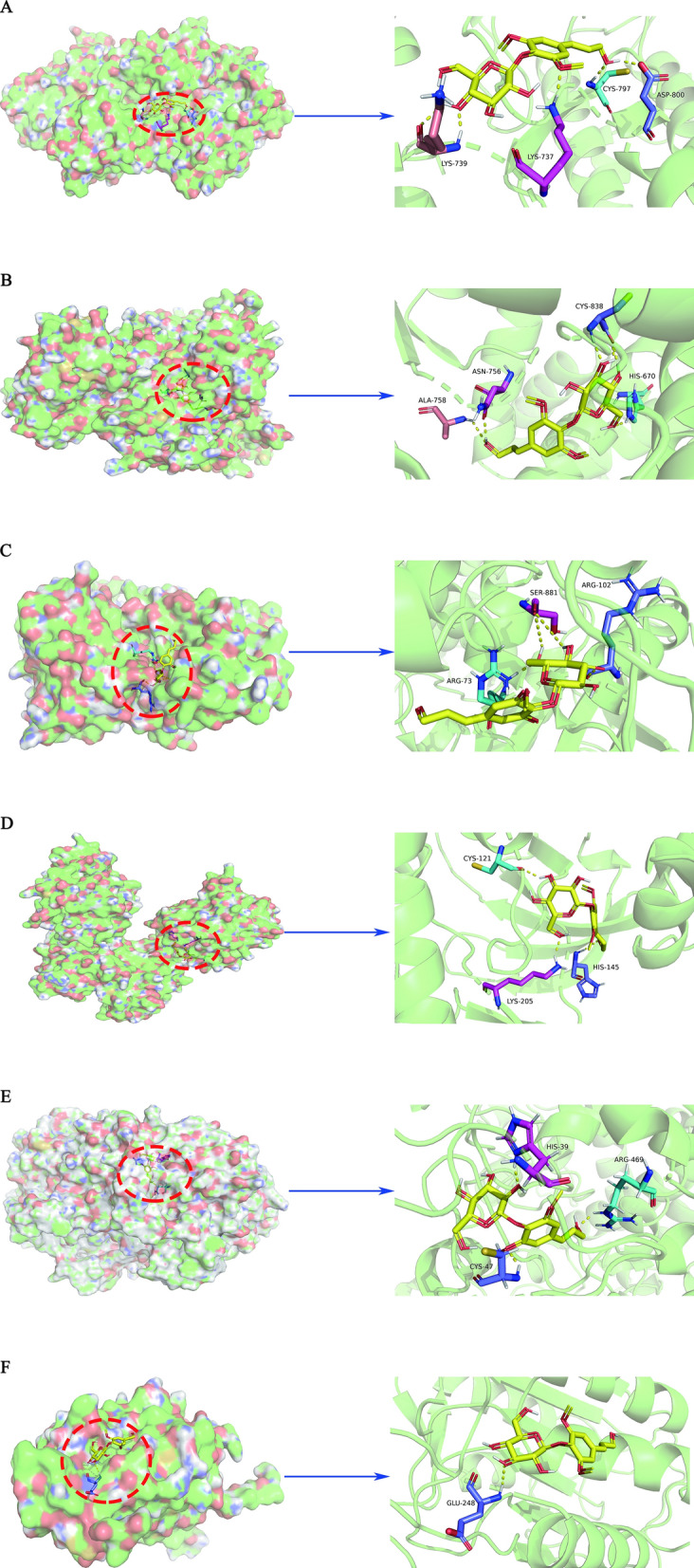
Docking results between Syringin and genes. **A** 6DUK. **B** 3ZIM. **C** 7JHP. **D** 3VVH. **E** 5F19. **F** 2H5I

It was clear to find that the hydroxyl group on glycosides had a strong binding ability with genes, and LYS, ASP, and CYS residues in many genes could effectively bind to Syringin. Therefore, all of them were the focus of our further study. A comprehensive analysis of the binding amino acid residues in PDB and the above results showed that there were some similarities between them. Among the first to fifth targets, A: HIS 39 in PTGS2 (COX-2, 5F19), A: SER 881 in HRAS (7JHP), A: ASP 800 in EGFR (6DUK), A: CYS 838 in PIK3CA (3ZIM), B: HIS 145 and B: CYS 121 in MAP2K1 (ERK1, 3VVH) all bind to small molecules in PDB database and Syringin. And the above amino acid residues had been verified by experiments, which suggested that Syringin could bind to these amino acid residues and play an inhibitor role in regulating the expression of these 5 genes. Although there were no such amino acid residues in Caspase 3 (2H5I), the function of the binding sites between Syringin and Caspase 3 was worthy of further study. The corresponding information is shown in Fig. 3.

### Two genes significantly related to survival and prognosis of BC patients

The differentially expressed genes in BC and normal tissues may be closely related to the survival rate of BC patients. Therefore, bioinformatics tools were used to analyze whether these hub targets could be developed as BC biomarkers.

**Fig. 4:**
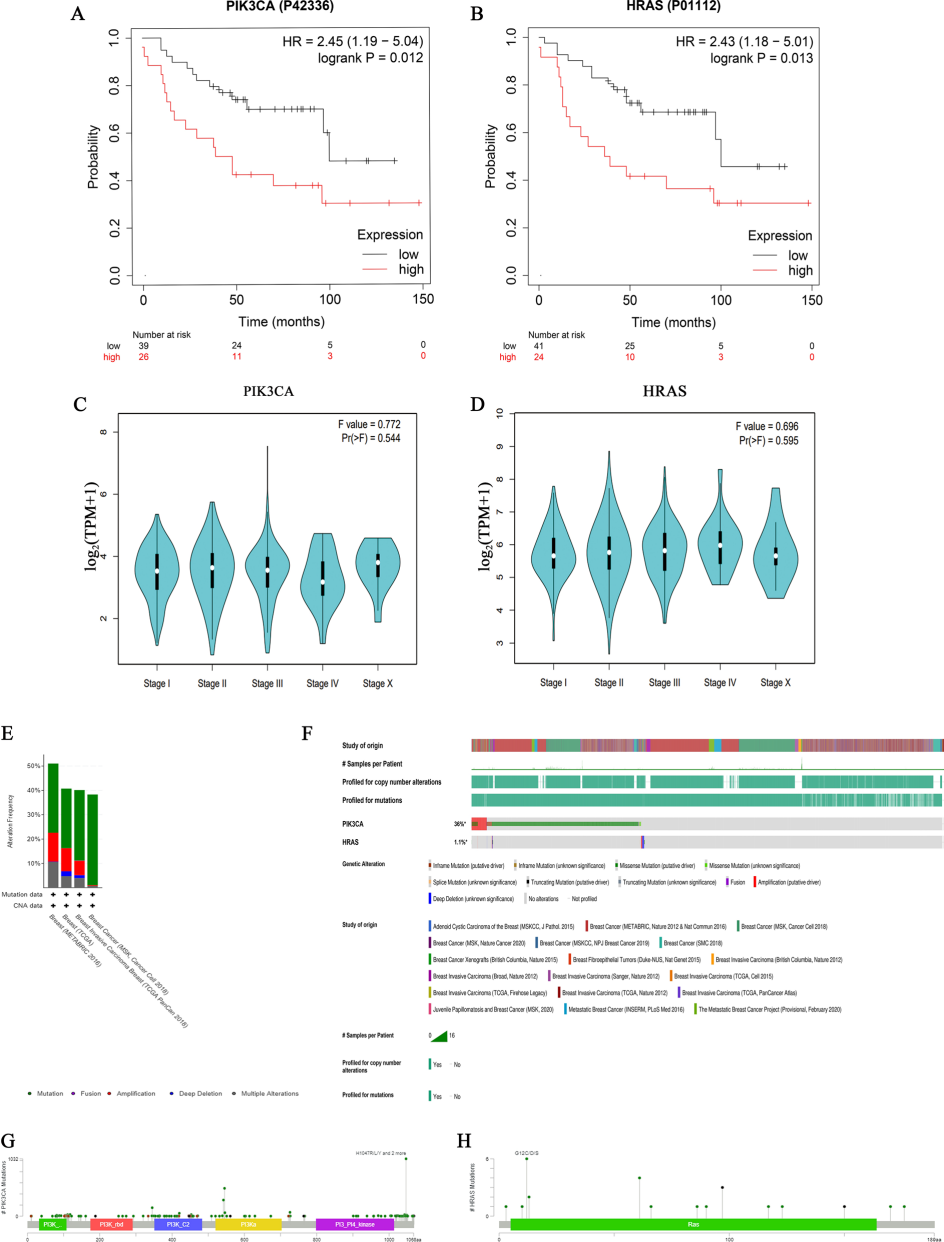
Biomarkers and their mutation in BC. Relationship between PIK3CA (**A**), HRAS (**B**), and survival rate and prognosis of BC patients. **C** Relationship between PIK3CA and stages of BC. **D** Relationship between HRAS and stages of BC. **E** Their mutation rates in four major studies. **F** Their specific mutation rates in 18 studies. **G** Mutation sites of PIK3CA. **H** Mutation sites of HRAS

The results of the Kaplan Meier plotter showed that in 65 clinical samples, high expression of PIK3CA (HR = 2.45(1.19–5.04), logrank P = 0.012) and HRAS (HR = 2.43(1.18–5.01), logrank P = 0.013) significantly reduced the survival rate of BC patients (Fig. 4A and B). Although the expression of PIK3CA and HRAS changed in different stages of BC, there was no significant correlation in GEPIA 2.0 (Fig. 4C and D). The mutation of the pathogenic gene is one of the main causes of tumorigenesis. PIK3CA and HRAS, as common mutational oncogenes, were found to have a high mutation rate in many tumors. By analyzing 9536 BC samples from 18 studies in the cbioPortal database, the combined mutation rates of them were found to be significantly high in 4 large sample studies (Fig. 4E). In 9536 samples, the mutation rate of PIK3CA and HRAS was 36% and 1.1%, respectively (Fig. 4F). This suggested that the mutation of HRAS was not the main reason for BC development. But the high mutation rate of PIK3CA was closely related to its promoting effect on BC. H1047R/L/Y and G12C/D/S were the most easily mutated sites of PIK3CA and HRAS, respectively (Fig. 4G and H). But the mutation rate and site of them are needed to be confirmed in more clinical samples.

### Construction of “mRNA-miRNA-lncRNA” network

**Fig. 5:**
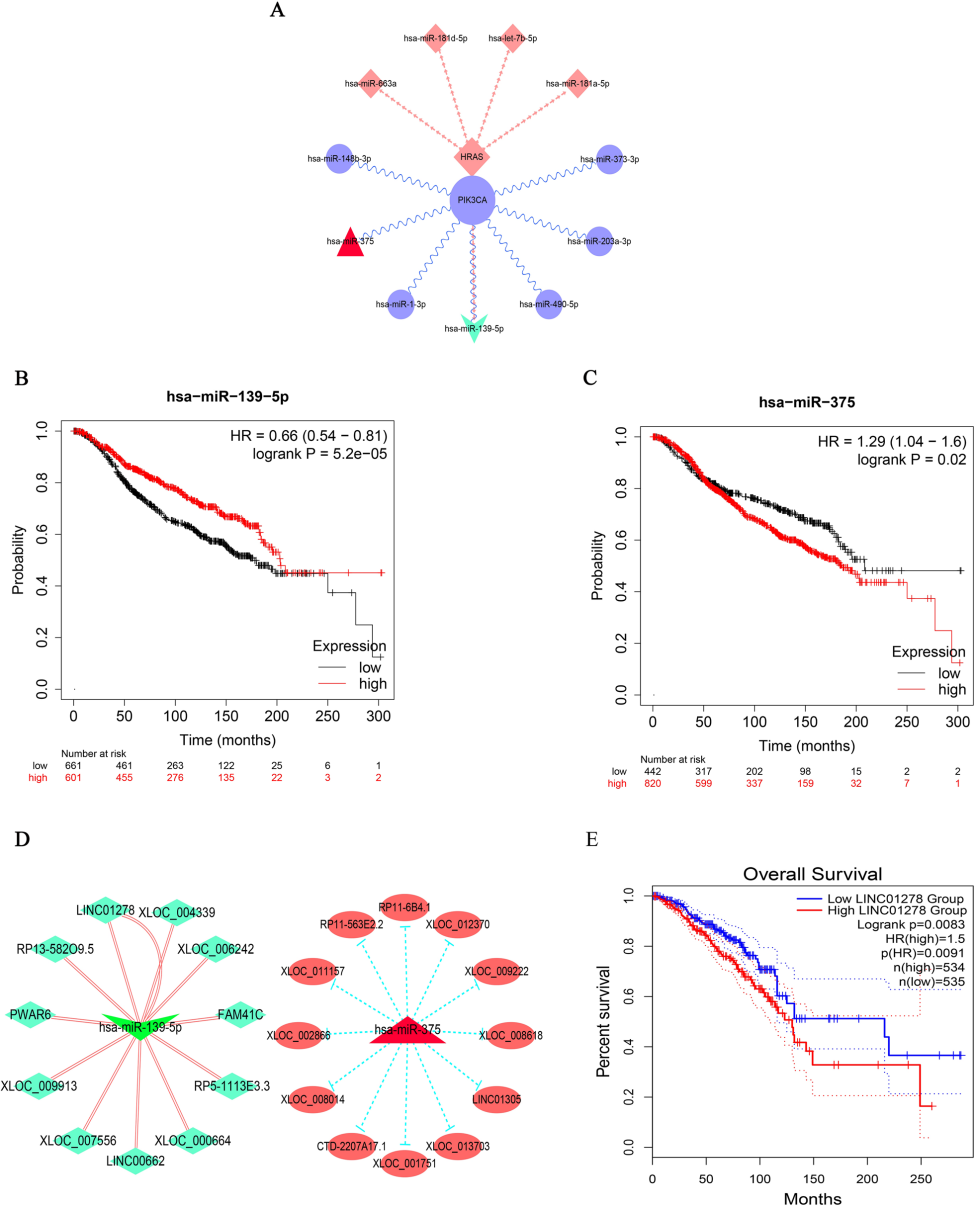
Results of miRNA and lncRNA analysis. **A** A mRNA-miRNA network. Relationship between hsa-miR-139-5p (**B**), hsa-miR-375 (**C**), and survival rate and prognosis of BC patients. **D** lncRNAs associated with hsa-miR-139-5p and hsa-miR-375. **E** Relationship between LINC01278 and survival rate and prognosis of BC patients

GO enrichment results showed that these targets were involved in gene silencing by miRNA. Therefore, bioinformatics databases were used to further study the effect of PIK3CA and HRAS on the occurrence and development of BC at the molecular level. MiRTarBase and miRWalk were used to obtain 7 miRNAs related to PIK3CA and 4 miRNAs related to HRAS (Fig. 5A). The results of Kaplan Meier Plotter and GEPIA 2.0 showed that the low expression of hsa-mir-139-5p (HR = 0.66 (0.54–0.81), log rank P = 5.2e-05) and the high expression of hsa-mir-375 (HR = 1.29 (1.04–1.6), log rank P = 0.02) were associated with low survival rate and poor prognosis in BC patients (Fig. 5B and C). Using TarBase v.8 and lnCAR, 11 lncRNAs associated with hsa-mir-139-5p and 12 lncRNAs associated with hsa-mir-375 were obtained (Fig. 5D and E). The high expression of LINC01278 (HR = 1.5 (1.08–2.09), logrank P = 0.016) was associated with a low survival rate and poor prognosis of BC patients (Fig. 5F). These miRNAs and lncRNAs related to PIK3CA and HRAS will lay a foundation for our further study.

### Syringin inhibits the proliferation of MDA-MB-231 and MCF-7 cells via the RAS-RAF-MEK-ERK pathway

**Fig. 6:**
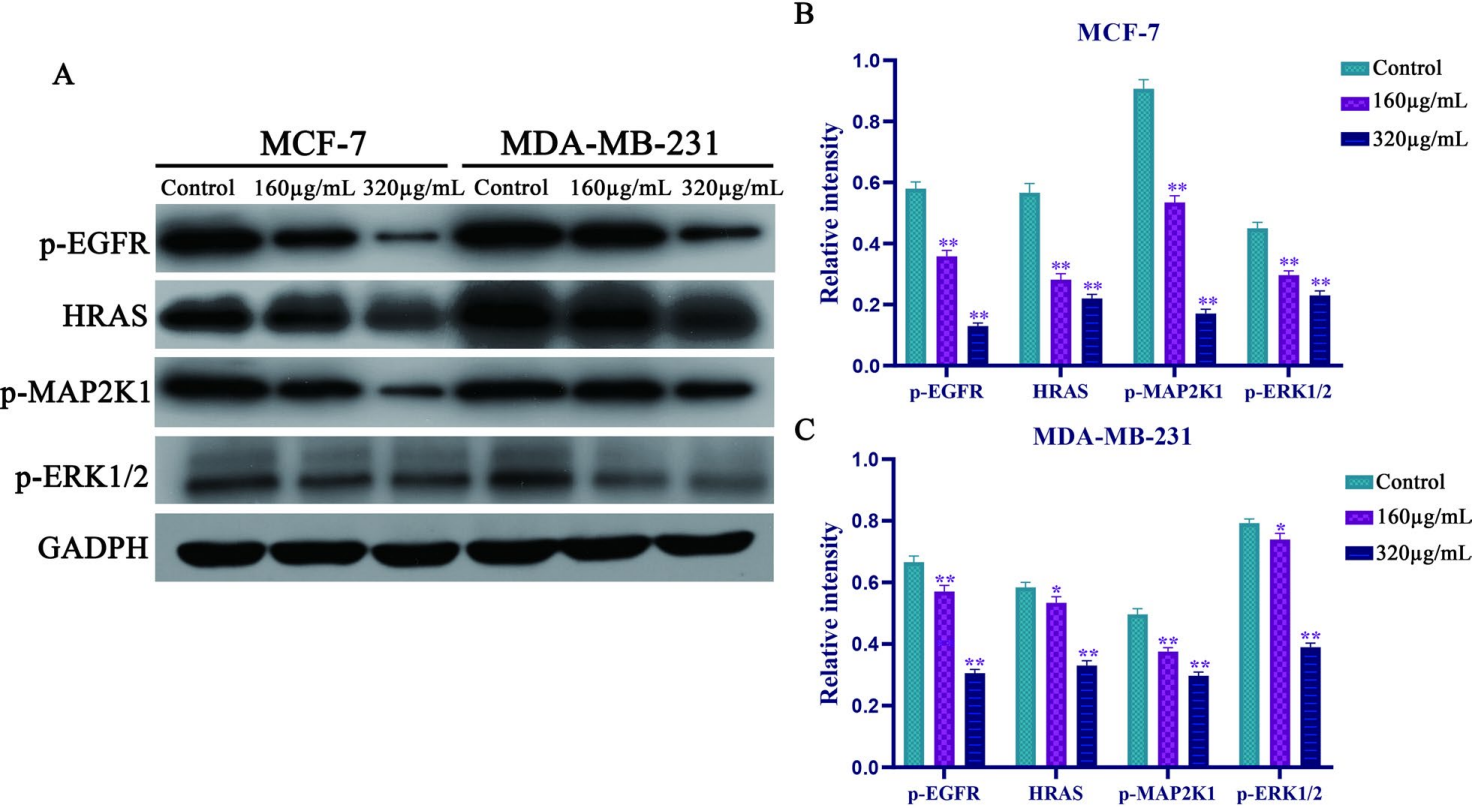
**A** The expressions of p-EGFR, HRAS, p-MAP2K1, and p-ERK1/2. **B** Syringin decreased their expression of them in MCF-7 cells. **C** Syringin decreased their expression of them in MDA-MB-231 cells. *P < 0.05 and **P < 0.01

As the western blot results showed that compared with the control groups, the expressions of p-EGFR, p-HRAS, p-MAP2K1, and p-ERK1/2 in both of them were significantly decreased in different concentrations of Syringin groups (P < 0.05 or P < 0.01) (Fig. 6A–C). Based on the literature and KEGG results, the above genes can promote the proliferation of tumor cells. Thus, Syringin inhibited their proliferation of them by regulating the RAS-RAF-MEK-ERK pathway.

### Syringin promoted apoptosis of MDA-MB-231 and MCF-7 cells in vitro

**Fig. 7:**
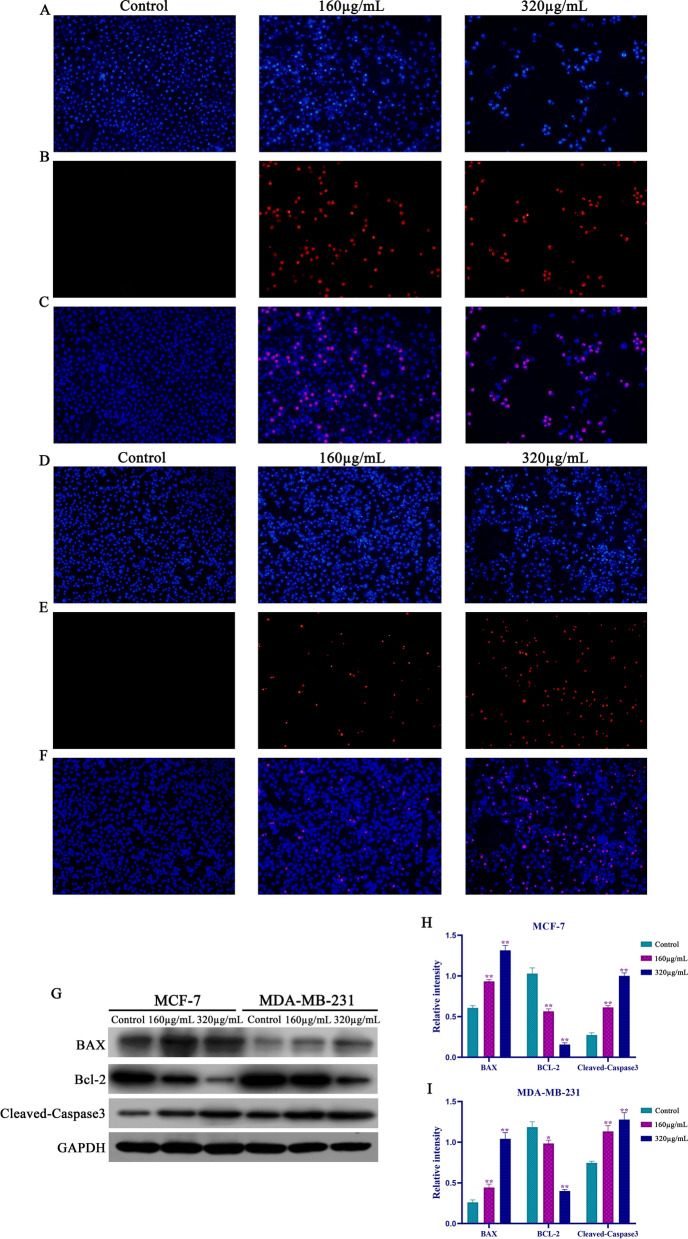
Syringin increased the necrotic and apoptosis of MCF-7 cells (**A**–**C**) and MDA-MB-231 cells (**D**–**F**). **G** The expression of BAX, Bcl-2, and Cleaved-Caspase3. **H**–**I** Syringin increased the expression of BAX and Cleaved-Caspase3 and decreased the expression of Bcl-2. *P < 0.05 and **P < 0.01

To determine whether Syringin promoted MDA-MB-231 and MCF-7 cells’ apoptosis, the apoptotic situation was examined by Hoechst 33,342/PI staining. Compared with the control groups, early apoptosis in the MCF-7 treated with Syringin significantly increased in Hoechst staining (Fig. 7A). PI staining results and the merged images showed that the most of apoptosis MCF-7 cells treated with Syringin were necrosis or late apoptosis cells (Fig. 7B, C). Interestingly, MDA-MB-231 cells treated with different concentrations of Syringin had more apoptotic cells but lower apoptosis rate than MCF-7 treated with Syringin (Fig. 7D). PI standing results and the merged images showed that the proportion of necrotic or late apoptotic cells in apoptotic cells was lower than that of MCF-7 cells treated with different concentrations (Fig. 7E and F). Therefore, Syringin is more likely to promote necrosis or late apoptosis of MCF-7 cells than MDA-MB-231 cells. However, more experiments are needed to study the specific period of apoptosis of BC cells after treatment with Syringin.

The expression of apoptosis regulators Bcl-2, BAX, and Cleaved-Caspase3 in MDA-MB-231 and MCF-7 cells were further studied by Western blot analysis. As Fig. 7G–I, the expression of BAX and Cleaved-Caspase3 in the Syringin group increased significantly, while the expression of Bcl-2 decreased (*P* < 0.05 or *P* < 0.01). And the expression changes of the above proteins in MCF-7 cells treated with Syringin were greater than those in MDA-MB-231 cells treated with Syringin, which was consistent with the Hoechst 33,342/PI staining results.

### Syringin inhibited the migration of MDA-MB-231 and MCF-7 cells through the PI3K-AKT-COX-2 pathway

**Fig. 8:**
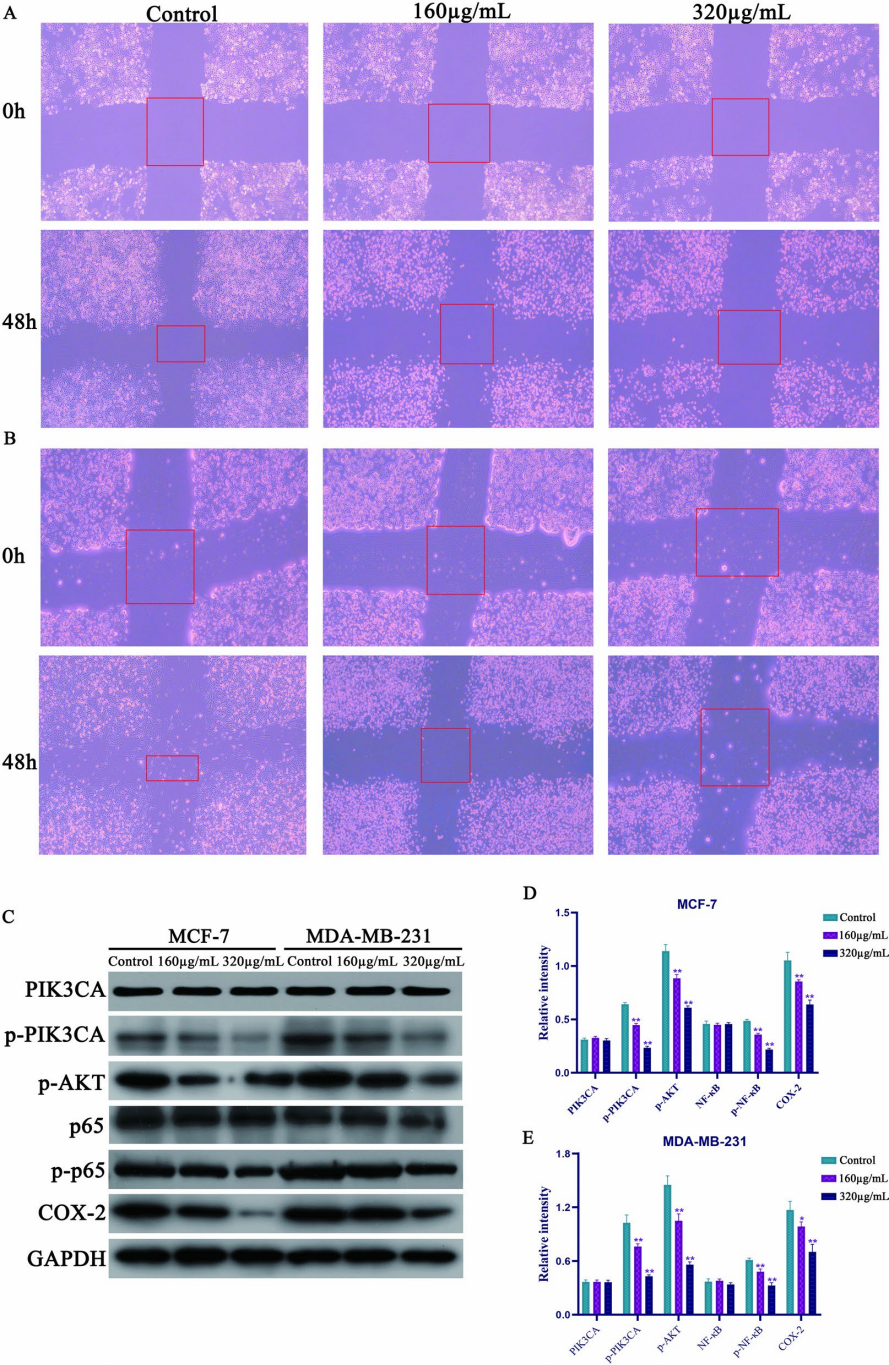
Syringin decreased the migration of MCF-7 cells (**A**) and MDA-MB-231 cells (**B**). **C** The expression of PIK3CA, p-PIK3CA, p-ATK, p65, p-p65, and COX-2. **D** Syringin decreased the expression of p-PIK3CA, p-ATK, p-p65, and COX-2, but didn't change the expression of PIK3CA and p65 in MCF-7 cells. **E** Syringin decreased the expression of p-PIK3CA, p-ATK, p-p65, and COX-2, but didn't change the expression of PIK3CA and p65 in MDA-MB-231 cells. *P < 0.05 and **P < 0.01

The inhibitory effect and mechanism of Syringin on the migration of these two cell lines were investigated by cell migration and western blot assays. As shown in Fig. 8A and B respectively, after 48 h, the area of the red rectangle decreased in each control group but decreased slowly with the increase of Syringin concentration.

The results of the western blot showed that the expressions of p-PIK3CA, p-AKT, p-NFκB (p-p65), and PTGS2 (COX-2) were significantly decreased in Syringin groups (*P* < 0.05 or *P* < 0.01). But there were no significant differences in the expression of total PIK3CA and NFκB (p65) between groups. The specific results are shown in Fig. 8C–E. It is worth noting that the scratch test is just a simple mean of presenting the migration trend, and it has certain limitations. Therefore, the establishment of this conclusion needs to be verified by more in vitro and in vivo experiments.

### Syringin down-regulated the expression of PIK3CA and HRAS

**Fig. 9:**
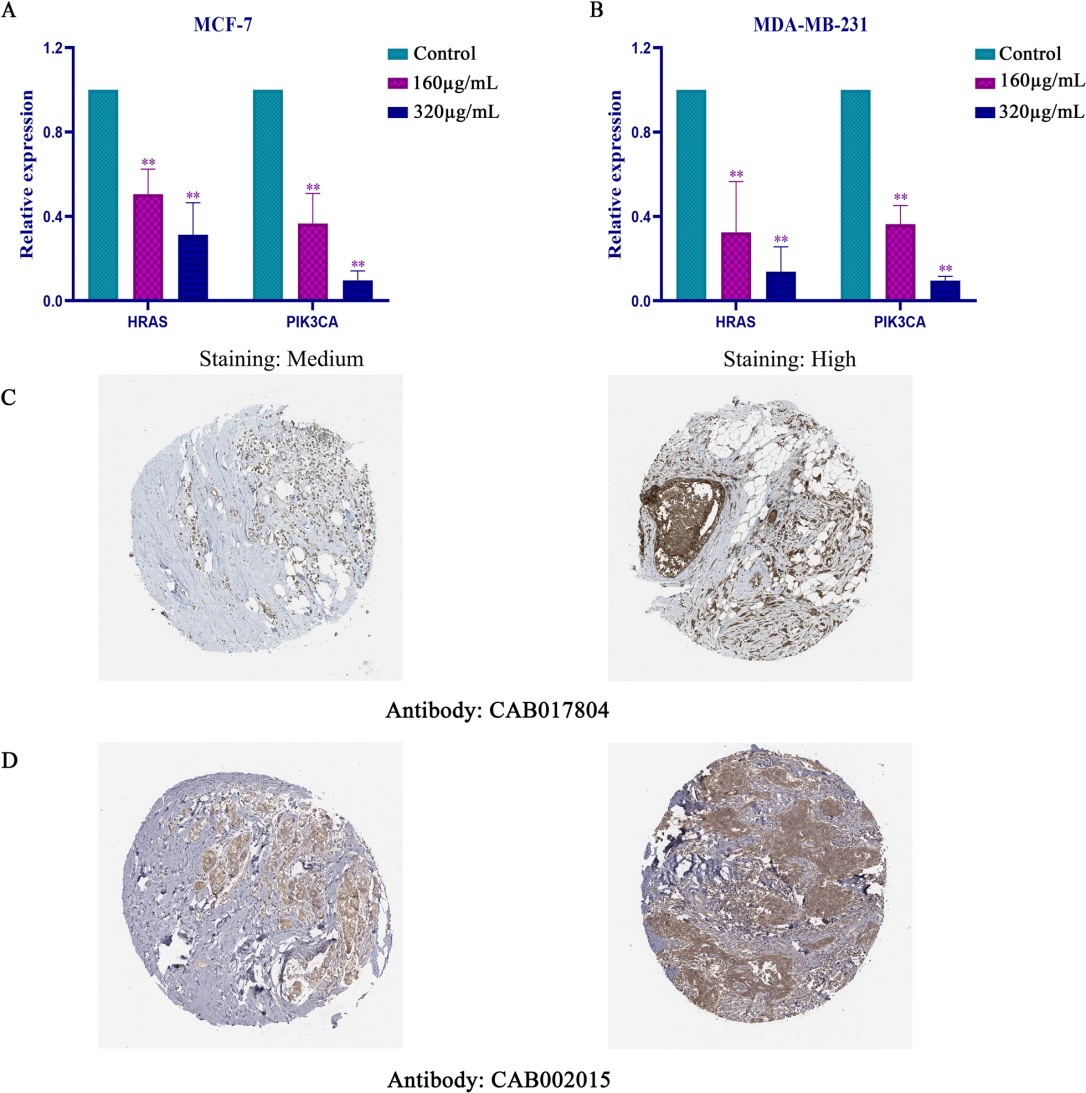
The expression of PIK3CA and HRAS by Real-Time PCR. **A** Syringin decreased their expression in MCF-7 cells. **B** Syringin decreased their expression in MDA-MB-231 cells. *P < 0.05 and **P < 0.01. **C** The expression of PIK3CA in normal breast and BC tissues. **D** The expression of HRAS in normal breast and BC tissues

Using the Human Protein Atlas database to analyze the pathology atlas of 12 BC patients in antibody:CAB017804, it was found that all patients had moderate or high levels of PIK3CA protein expression. And in antibody:CAB002015, 7 of 12 BC patients had moderate or high levels of HRAS protein expression (Fig. 9C, D). Our previous western blot results confirmed that different concentrations of Syringin significantly reduced the expression of PIK3CA and HRAS in BC. And Real-time PCR results showed that it can significantly down-regulate the expression level of PIK3CA and HRAS (Fig. 9A, B). But their roles in the therapeutic effect of Syringin on these two BC cell lines need to be confirmed by more experiments.

## Discussion

Breast cancer is the leading cause of cancer death in women worldwide. Until now, no effective and low-toxic drugs for the treatment of BC have been discovered. For a long time, TCM has played a significant role in the treatment of tumors in China with the advantage of mild side effects [33]. Therefore, it has become a new trend to develop effective anti-tumor natural components from TCMs.

Relevant studies [34] have shown that the methanol extract of ASH has a certain inhibitory effect on some cancers and many components of it are used in a variety of drugs. Compound cantharidin capsule has been mainly used for the treatment of rectal cancer, lung cancer, malignant lymphoma, primary liver cancer, and gynecological malignant tumor [35]. Wei-da-kang oral liquid shows significant therapeutic effects on leukopenia, thrombocytopenia, and immune function decrease caused by tumor radiotherapy and chemotherapy. Syringin, as one of the main components of these two kinds of drugs, the current research only focuses on the immune-enhancing and anti-inflammatory effects of it [36, 37]. Therefore, this study aims to study the effect and mechanism of Syringin against BC via network pharmacology, molecular docking, bioinformatics, and in vitro experiments.

The results of MTT showed that Syringin had a time- and dose-dependent inhibitory effect on the growth of MDA-MB-231 and MCF-7 cells. MAP2K1, PIK3CA, HRAS, EGFR, Caspase 3, and PTGS 2 were the hub targets of Syringin for the treatment of them based on network pharmacology results. Molecular docking demonstrated that Syringin bound to these hub targets at the confirmed amino acids, excluding Caspase 3 (2H5I).

**Fig. 10:**
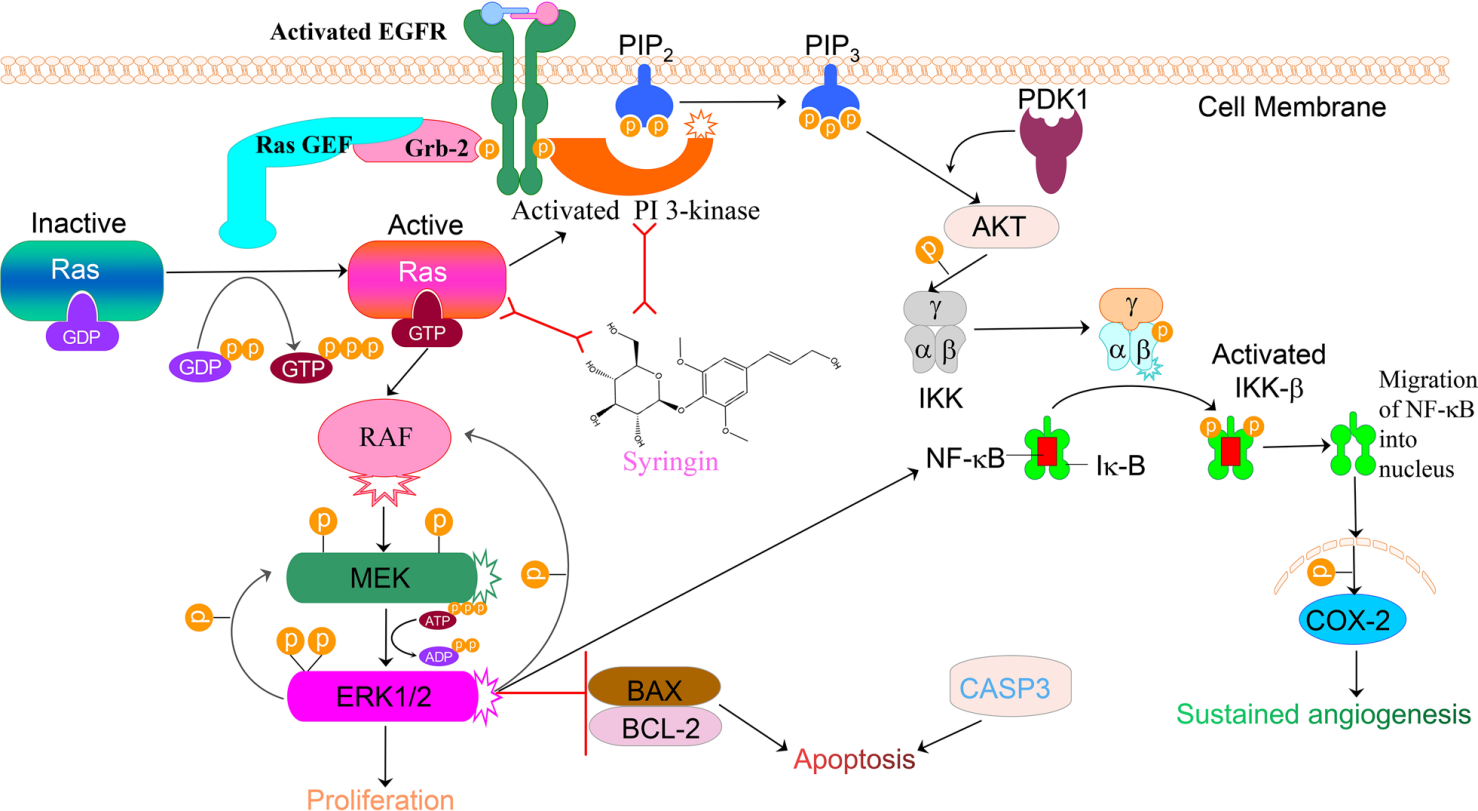
The mechanism of Syringin against BC

EGFR is a transmembrane receptor in the receptor tyrosine kinase family. It forms a dimer by binding with growth factors, which phosphorylates the tyrosine sites in its intracellular region, and then regulates the expression of RAS and PI3K [38]. When the receptor tyrosine kinase is activated, RAS is converted from the inactivated state of GDP binding to the activated state of GTP binding by the RAS guanylate exchange factor (GEF). As a subtype of RAS, HRAS is responsible for encoding and producing HRAS protein, which transmits extracellular signals to the nucleus to participate in the above processes and regulate cell proliferation, maturation, and differentiation [39]. PIK3CA is a subtype of PI3K, which encodes P110 α protein. In tumor cells, AKT is prone to mutate and continuously stimulates downstream genes, to increase the ability of cell invasion and metastasis [40]. RAS is known to regulate the expression of PI3K, which enhances the connection between the two pathways [41]. Based on the literature and above results, the 6 hub targets were found to be mainly involved in EGFR-RAS-RAF-MEK-ERK and PI3K-AKT-COX-2 pathways, which promoted the migration and proliferation and inhibited the apoptosis of tumor. The results of Hoechst 33,342-PI staining, plate cloning, cell migration, and western blot showed that Syringin significantly inhibited the proliferation and migration of MDA-MB-231 and MCF-7 cells and promoted their apoptosis via these two pathways. The probable mechanism of Syringin against them is shown in Fig. 10.

Bioinformatics is a valid method to explore the occurrence and development of diseases by using computer technology and clinical data. Through the analysis of 3951 clinical samples of BC, it was found that PIK3CA and HRAS were significantly associated with the survival and prognosis of BC patients, of which the expression changed moderately with the stages of BC. This suggested that they were potential biomarkers of BC, but the stage of BC could barely be diagnosed according to the expression of PIK3CA and HRAS. The accuracy of these results needs to be based on data from more clinical samples. Studies have shown that PIK3CA and HRAS are prone to a mutation in a variety of tumors, resulting in their persistent hyperactivity, which promotes cancer cell proliferation and migration [42, 43]. Therefore, this study analyzed 9536 BC samples from 18 studies via the cBioPortal database, and the results showed that the mutation rate of PIK3CA was 36%, while that of HRAS was only 1.1%. This suggested that PIK3CA mutation was closely related to the occurrence and development of BC. However, the mutation does not influence the effect of HRAS on BC. Therefore, PIK3CA might be regulated by EGFR and HRAS in the case of its mutation, thus enhancing the stimulation of downstream cells. At present, the commonly used inhibitors of PIK3CA and HRAS are mainly divided into two categories [44]. The first one is to prevent the expression of downstream proteins by inhibiting their related kinases. The second one is to directly bind with the pockets of PIK3CA and HRAS to inhibit their functions. Molecular docking results suggested that Syringin bound with amino acid sites A: Cys 838 on PIK3CA and A: ASP 119 on HRAS which have been confirmed to bind with other small molecules to inhibit the expression of PIK3CA and HRAS. The expression levels of these two proteins in 12 BC patients in the Human Protein Atlas database and the results of real-time PCR confirmed expression of them in BC at protein and mRNA levels. This suggested that Syringin could be developed as their inhibitor. But more experiments were needed to confirm whether they could dock with Syringin.

lncRNAs can competitively bind to miRNAs, regulating the expression of proteins and affecting their biological function [45]. GO functional enrichment results showed that the hub targets participated in mRNA regulation of gene silencing. miRNAs and lncRNAs related to these two biomarkers could support our later research at the molecular level. 7 miRNAs related to PIK3CA and 4 miRNAs related to HRAS were obtained from multiple databases. Among them, hsa-mir-139-5p (HR = 0.66 (0.54–0.81), log rank P = 5.2 e-05) and hsa-mir-375 (HR = 1.29 (1.04–1.6), log rank P = 0.02) were correlated with the survival and prognosis of BC patients. Some studies have shown that the expression of hsa-mir-139-5p in human lung cancer cell lines inhibits the activity of DICER1, but does not inhibit PPP2R2A or LATS2. And it is a potential biomarker of squamous cell carcinoma (SCC) [46]. In addition, a platform was proved to be highly effective in the diagnosis of BC by analyzing the four tumor-related exons miRNA (mir-221, mir-375, mir-1246, and mir-21) in the BC cohort (AUC: 0.989) [47]. Barbara Zellinger also demonstrated that hsa-mir-375/RASD1 signal pathway could predict the condition of early BC patients [48]. And it could also inhibit stem cells of BC by targeting JAK2. LINC01278 suppressed the proliferation and apoptosis of OS cells by mediating miR-134-5p/KRAS axis, which was expected to be a potential therapeutic target for OS [49]. Patients with high expression of LINC01278 were poorly diagnosed. More studies have described that LINC01278 participated in multiple pathways to regulate colon and liver cancer [50, 51]. However, the relationship between the PIK3CA-hsa-mir-139-5p-LINC01278, PIK3CA-hsa-mir-375, and BC has not been elaborated in detail, which is required to be explored. And they might be closely related to the therapeutic effect of Syringin on these two BC cell lines.

Interestingly, Lee et al. confirmed that syringin inhibited the growth of breast carcinoma cells by up-regulation p21, cleaved caspase-3/caspase-9 and PARP, and down-regulation CDK4 and XIAP [14]. It also caused high levels of ROS in breast cancer cells. These results suggest that Syringin has potential to be an effective agent to treat breast cancer through the elevation of ROS. Based on bioinformatics, molecular docking and in vitro experiments, this study further proved the possible anti-BC effect of Syringin and its mechanism of action. Nevertheless, several experiments are required in future studies for verification. Firstly, the expression of the above proteins and their relationship with the prognosis and survival of BC patients should be explored in more clinical samples. Secondly, more in vitro and in vivo experiments should be used to verify the relationship between Syringin, PIK3CA-hsa-miR-139-5p-linc01278, PIK3CA-hsa -miR-375, and BC, to clarify its effect and mechanism on MDA-MB-231 and MCF-7 cells.

## Conclusions

Taken together, this study confirmed that Syringin inhibited the proliferation and migration of MDA-MB-231 and MCF-7 cells and promoted their apoptosis by regulating EGFR-RAS-RAF-MEK-ERK and PI3K-AKT-COX-2 pathways via network pharmacology, molecular docking, and in vitro experiments. Meanwhile, bioinformatics analysis showed that PIK3CA and HRAS were two potential biomarkers of BC, and the PIK3CA-hsa-mir-139-5p-LINC01278 and PIK3CA-hsa-mir-375 pathways were closely related to the effect and mechanism of Syringin on BC. But more in vitro and in vivo experiments should be used to validate these results.

## Supplementary Information

**Additional file 1.** The extraction, separation and identification of syringin.

## Data Availability

All data generated or analyzed during this study are included in this published article and are available on request to the corresponding authors.

## Abbreviations

BC: Breast cancer
TCM: Traditional Chinese medicine
ASH: Acanthopanax senticosus (Rupr. & Maxim.) Harms
MTT: 3-(4,5)-Dimethylthiahiazo (-z-y1)-3,5-di- phenytetrazoliumromide
ADMET: Absorption, Distribution, Metabolism, Excretion, and Toxicity
DMSO: Dimethyl sulfoxide
C-T-P: Components-targets-pathways
PPI: Protein–protein interaction
TLC: Thin-layer chromatography
LC–MS: Liquid chromatography-mass spectrometry
HPLC: High-performance liquid chromatography
NMR: Nuclear magnetic resonance
TCMSP: Traditional Chinese medicine systems pharmacy database and analysis platform
DMEM: Dulbecco's modified eagle medium
FBS: Fetal bovine serum
DAVID: Database for annotation, visualization and integrated discovery
KEGG: Kyoto Encyclopedia of Genes and Genomes
GO: Gene Ontology
PI: Propidium iodide
RIPA: Radio-immunoprecipitation assay
PMSF: Phenylmethanesulfonyl fluoride
GEPIA: Gene expression profiling interactive analysis
GEO: Gene expression omnibus
TCGA: The cancer genome atlas
GTEX: Genotype-tissue expression
Caco-2: Intestinal epithelial permeability
BBB: Blood–brain barrier
CNS: Central nervous system
IC 50: Half maximal inhibitory concentration
