# Supplementary material for: Syringin exerts anti-breast cancer effects through PI3K-AKT and EGFR-RAS-RAF pathways
Source: J Transl Med. 2022 Jul 6;20:310. doi: 10.1186/s12967-022-03504-6 (PMC9258109; doi:10.1186/s12967-022-03504-6)
Supplement: Supplementary file 1 — Additional file 1. The extraction, separation and identification of syringin. [file 12967_2022_3504_MOESM1_ESM.docx]

**The extraction, separation and identification of syringin**

**Fei Wang^1^, Chong Yuan^1^, Bo Liu^1,2*^, Yan-Fang Yang^1,2*^, He-Zhen Wu^1,2*^**

^1^Faculty of Pharmacy, Hubei University of Chinese Medicine, Wuhan 430065, China

^2^Key Laboratory of Traditional Chinese Medicine Resources and Chemistry of Hubei Province, Wuhan 430061, China

*** Correspondence:**Bo Liu
4860715@qq.com

He-Zhen Wu
hezh_wu@163.com

Yan-Fang Yang
yyf0204@hbtcm.edu.cn

*Acanthopanax senticosus* (Rupr. & Maxim.) Harms (ASH) has a pungent taste, which tastes bitter and slightly sweet, and its character is pungent and warm. It is first recorded in *Shennong’s Classic of Materia Medica* and has a variety of clinical effects. In China, it has been used as medicine for thousands of years. Modern pharmacological research has found that AS has a significant anti-cancer effect. Therefore, *MTT* test was used to investigate the toxicity to MCF-7, and HPLC was used to analyze the chemical components of the effective part. And the component with the highest content in the target part was extracted and its structure were identified by nuclear magnetic resonance (NMR), liquid chromatograph-mass spectrometer (LC-MS) and high performance liquid chromatograph (HPLC).

# Materials and methods

## Screening of target component

The ASH was collected from Wuhan Hubei. The plants were certified by professor He-Zhen Wu, Hubei University of Chinese Medicine. It is stored in Hubei Key Laboratory of traditional Chinese medicine chemistry and traditional Chinese medicine resources, Hubei University of Chinese medicine. The AS (1.0 kg) was ground and soaked in 75% ethanol (3.0 L) for 24 h. Then, 7.0 L 75% ethanol were added for infiltration. The combined ethanol extracts were concentrated by BUCHI Rotary evaporator (R-300 series).

Then, MCF-7 cells were cultured at a density of 6.0×10^3 cells per well in 96 well plates. After cell adhesion, plates were injected with ethanol extract at different concentrations (0, 20, 40, 80, 160 and 320 µg/mL) for 24 h respectively. Then, 20 µL *MTT* (sigma) was added to each well and the cells were cultured for another 4 h. After removing the supernatant, 150 µL dimethyl sulfoxide (DMSO) (sigma) was added to each well and stirred on QB-9001 micropore rapid shaker (Kylin-Bell Lab Instruments Co. Ltd., Jiangsu, China) for 10 minutes to obtain crystal violet product. Finally, the absorbance of each well was read at 490 nm using spark 10 m microplate reader (Tecan, Männedorf, Switzerland).

## 2. Preparation of Syringin

In order to further determine the effective components of AS, it was separated and purified by systematic solvent extraction, followed by petroleum ethersequentially extracted with Petroleum ether, Ethyl acetate and n-Butanol respectively. Based on our group preliminary study, the n-butanol part was selected as the target. A gradient system of chloroform-methanol（50: 1 ~ 40: 1 ~ 30: 1 ~ 20: 1 ~ 10: 1 ~ 1: 1, v/v）and Thin-layer chromatography (TLC) were used to yield 11 subfractions(A-K). Fraction F was subjected to column chromatography and eluted with a gradient system of chloroform-methanol acetate (15: 1, v/v) to yield the target component. Sephadex gel column for further separation and purification were used to obtain it. The purity and molecular weight of this component were checked by 1260 HPLC (Agilent, CA, USA), 600 MHz NMR and Xevo G2-XS QTOF mass spectrometer (Waters, MA, USA), respectively.

## 2.1 HPLC analysis

Chromatographic separation was performed using a column of Waters ACQUITY UPLC BEH C18 (100 × 2.1 mm, 1.7 µm) at 30 °C and the injection volume was set to 3 µl. Detection was at 268 nm and the mobile phase consisted of ultrapure water (A) and acetonitrile (B). The following gradient elution program was used: 0-15 min, 10-30% B; 15-45 min, 30-90% B; 45-50 min, 90% B.

## 2.2 NMR analysis

The purified samples were sent to the Institute of physics and mathematics, Wuhan Chinese Academy of Sciences for NMR identification.

## 2. 3 LC-MS analysis

The mass spectrometer equipped with a source of electrospray ionization (ESI) operating in positive mode. The capillary voltage of 3 kV, desolvation gas flow of 600

# Results

## 1. *Analysis of target component*

The *MTT* results showed that the ethanol extract of ASH significantly decreased the growth of MCF-7 cells, as shown in the Figure S1.

According to the literatures, ASH has been reported to inhibit some tumors for many times, however, there were few studies on BC. Based on the results and literatures, we found that its marker component Syringin was also effective for BC. Therefore, varieties of extraction and separation methods were used to purify and identify this component.


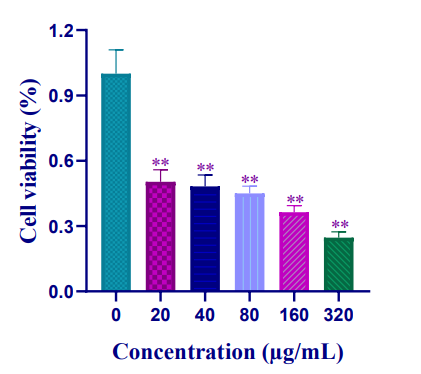


**Supplementary Figure 1.** The *MTT* result of ASH.

## 2.1 HPLC analysis

The obtained white crystal (50.1 mg) was easily soluble in methanol. The mixture of component and reference substance showed only one peak at corresponding time (Figure S2 A) and its purity was more than 98.5% by HPLC (Figure S2B).


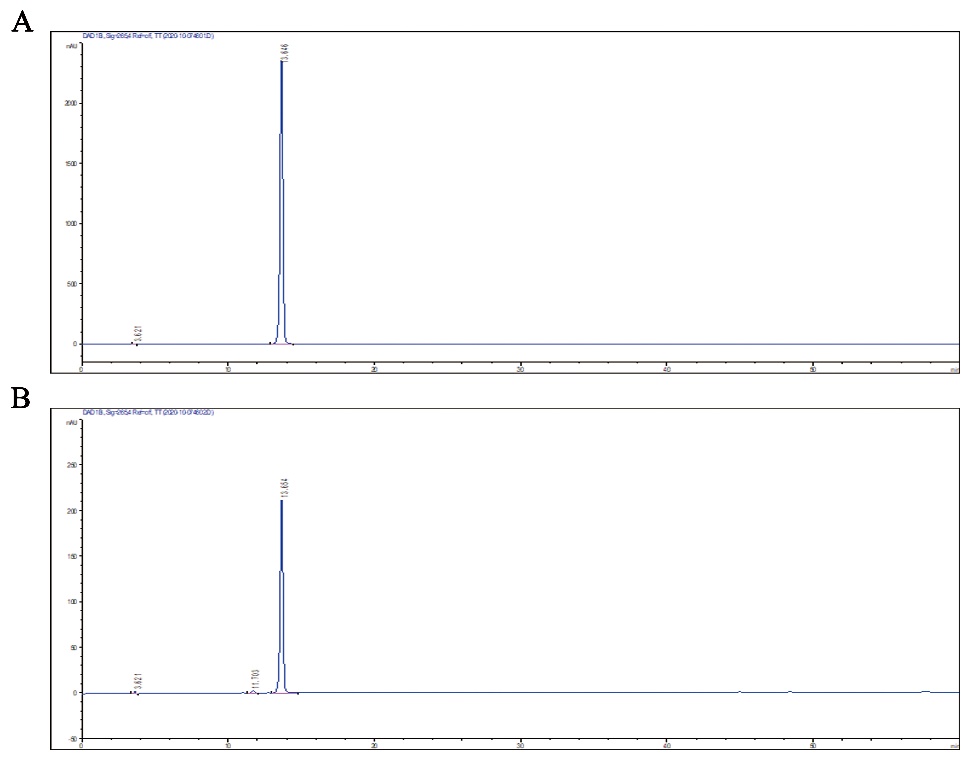


**Supplementary Figure 2.** A: HPLC chromatograms of the sample mixed with Syringin reference substance 1:1; B: HPLC chromatograms of the sample.

## 2.2 NMR analysis

After systematic analyzing H-spectrum data, C-spectrum, C-H correlation and H-H correlation data in NMR, we identified the component 1 as Syringin. Furthermore, it was compared with the data recorded in the literature.

The specific results of the analysis are as follows: 1H NMR (600 MHz, Methanol-d4) δ 6.66 (s, 2H), 6.45 (dt, J = 15.9, 1.6 Hz, 1H), 6.23 (dt, J = 15.9, 5.6 Hz, 1H), 4.76 (d, J = 18.5 Hz, 67H), 4.49 (s, 5H), 4.13 (dd, J = 5.6, 1.6 Hz, 2H), 3.76 (s, 6H), 3.68 (dd, J = 11.9, 2.4 Hz, 1H), 3.59 – 3.53 (m, 1H), 3.41 – 3.28 (m, 3H), 3.25 (s, 3H), 3.11 (ddt, J = 7.5, 5.2, 2.4 Hz, 1H), 1.30 – 1.10 (m, 1H). According to the h-spectrum, it contains a 1,3,4,5-symmetric benzene ring. So, it contains 17 C totally (14 C in the c-spectrum). The proton signal of δ 4.13 (dd, J = 5.6, 1.6 Hz, 2H) indicates that there is a - CH2OH fragment in the structure. The proton signal of δ 3.76 (s, 6H) indicates that there are two methoxyl groups in the structure. In conclusion, the compound is supposed to be Syringin.


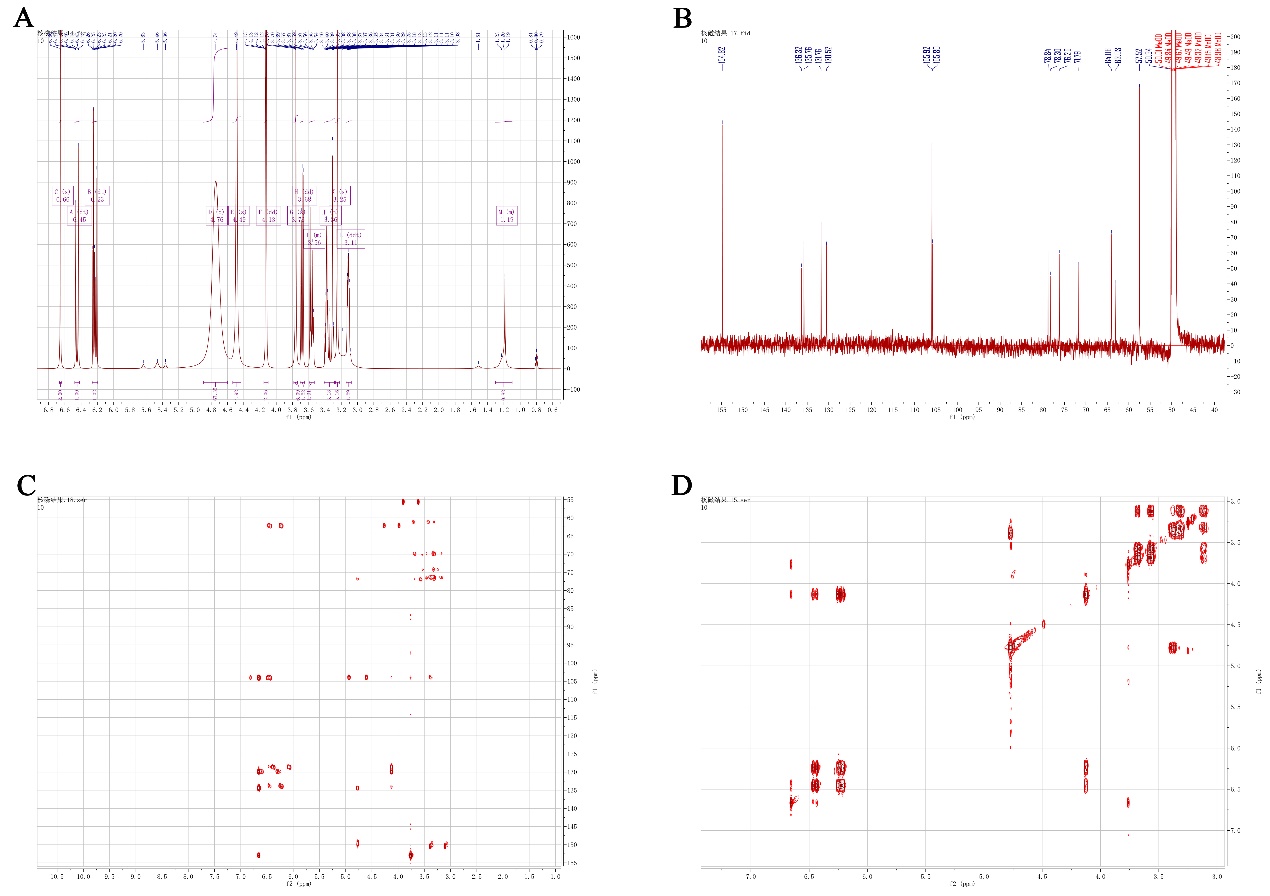


**Supplementary Figure 3.** A: H-spectrum; B: C-spectrum; C: C-H correlation; D: H-H correlation.

## 2.3 MS analysis

The total ion flow diagram and HPLC-UV chromatogram are shown in Figure S5 A. Mass spectrometry showed that its ESI-MS m/z 37.1 [M + Na] ^+^, 395.3 [M + Na] ^+^, which indicated that the molecular formula of this component was C10H8O4 (Figure S5 B-C). In summary, the white crystal is Syringin. As the landmark component of AS and the main active ingredient of a variety of listed drugs, health products and foods, such as wei-da-kang oral liquid, yan-li-xiao capsule, component cantharidin capsule, Syringin is mainly reported to have the effects of anti-inflammatory and enhancing immunity. At the same time, as one of the main active components in Aidi injection which has been used to against cancer, the inhibitory effect and mechanism of Syringin on breast cancer is not clear. It has certain research value. In conclusion, it is very meaningful to analyze the efficacy and mechanism of Syringin against breast cancer.


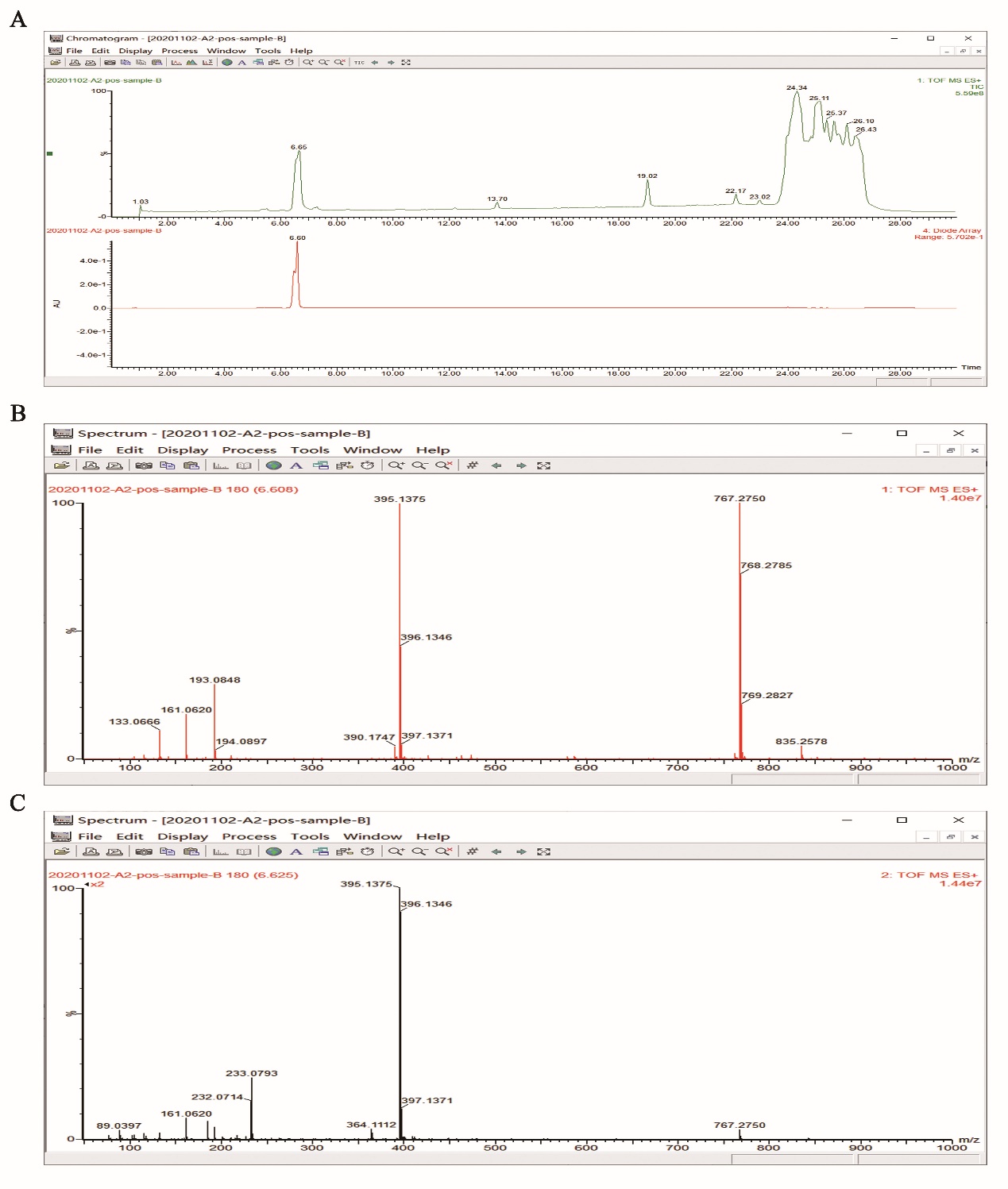


**Supplementary Figure 4.** A: Total ion chromatogram detected in the positive ion mode and HPLC-UV chromatogram obtained at 268 nm; B: A mass spectrometry; C: Two stage mass spectrometry.
